# Profiling the eicosanoid networks that underlie the anti- and pro-thrombotic effects of aspirin

**DOI:** 10.1096/fj.202000312R

**Published:** 2020-06-27

**Authors:** Marilena Crescente, Paul C. Armstrong, Nicholas S. Kirkby, Matthew L. Edin, Melissa V. Chan, Fred B. Lih, Jing Jiao, Tania Maffucci, Harriet E. Allan, Charles A. Mein, Carles Gaston-Massuet, Graeme S. Cottrell, Jane A. Mitchell, Darryl C. Zeldin, Harvey R. Herschman, Timothy D. Warner

**Affiliations:** 1 Centre for Immunobiology, Blizard Institute, Barts and the London School of Medicine and Dentistry, Queen Mary University of London, London, UK; 2 National Heart & Lung Institute, Imperial College London, London, UK; 3 Division of Intramural Research, National Institute of Environmental Health Sciences, Research Triangle Park, NC, USA; 4 Department of Medical and Molecular Pharmacology, David Geffen School of Medicine, University of California, Los Angeles, CA, USA; 5 Centre for Cell Biology and Cutaneous Research, Blizard Institute, Barts and the London School of Medicine and Dentistry, Queen Mary University of London, London, UK; 6 Centre for Endocrinology, William Harvey Research Institute, Barts and the London School of Medicine and Dentistry, Queen Mary University of London, London, UK; 7 Reading School of Pharmacy and ICMR, University of Reading, Reading, UK

**Keywords:** antithrombotic therapy, aspirin, endothelium, eicosanoid profiling, platelets

## Abstract

Aspirin prevents thrombosis by inhibiting platelet cyclooxygenase (COX)-1 activity and the production of thromboxane (Tx)A_2_, a pro-thrombotic eicosanoid. However, the non-platelet actions of aspirin limit its antithrombotic effects. Here, we used platelet-COX-1-ko mice to define the platelet and non-platelet eicosanoids affected by aspirin. Mass-spectrometry analysis demonstrated blood from platelet-COX-1-ko and global-COX-1-ko mice produced similar eicosanoid profiles in vitro: for example, formation of TxA_2_, prostaglandin (PG) F_2α_, 11-hydroxyeicosatraenoic acid (HETE), and 15-HETE was absent in both platelet- and global-COX-1-ko mice. Conversely, in vivo, platelet-COX-1-ko mice had a distinctly different profile from global-COX-1-ko or aspirin-treated control mice, notably significantly higher levels of PGI_2_ metabolite. Ingenuity Pathway Analysis (IPA) predicted that platelet-COX-1-ko mice would be protected from thrombosis, forming less pro-thrombotic TxA_2_ and PGE_2_. Conversely, aspirin or lack of systemic COX-1 activity decreased the synthesis of anti-aggregatory PGI_2_ and PGD_2_ at non-platelet sites leading to predicted thrombosis increase. In vitro and in vivo thrombosis studies proved these predictions. Overall, we have established the eicosanoid profiles linked to inhibition of COX-1 in platelets and in the remainder of the cardiovascular system and linked them to anti- and pro-thrombotic effects of aspirin. These results explain why increasing aspirin dosage or aspirin addition to other drugs may lessen antithrombotic protection.

## 1 | INTRODUCTION

Cardiovascular diseases are the leading cause of death and disability worldwide.^1^ They are associated with the formation of arterial thrombi following excessive platelet activation and aggregation at disrupted vascular sites resulting in acute vessel occlusion and ischemic events.^2,3^ Aspirin has been the cornerstone of antiplatelet therapy for more than three decades, with numerous trials demonstrating that low doses of aspirin reduce the incidence of secondary cardiovascular events.^4–6^ Recent studies have, however, raised doubts as to whether the previously observed benefits of aspirin in secondary prevention are maintained under current clinical care regimes where aspirin is often given together with a P2Y_12_ receptor antagonist, such as prasugrel or ticagrelor. For example, the platelet inhibition and patient outcomes (PLATO) trial reported an association between higher doses of aspirin and increased thrombotic risk in individuals taking ticagrelor.^7^ Newer studies are therefore evaluating the benefits of low- vs high-dose aspirin in patients who have had previous cardiovascular events^8^ and whether aspirin discontinuation in favor of single drug therapy with a P2Y_12_ receptor antagonist could retain antithrombotic efficacy.^9,10^ The first results of these trials indicate that low-dose aspirin does not increase the protection from ischemic events afforded by ticagrelor.^11,12^

The platelet inhibitory effects of aspirin are explained by its ability to irreversibly inhibit cyclooxygenase (COX)-1 in platelets, and therefore, the conversion of AA to thromboxane (Tx) A_2_, an eicosanoid that promotes platelet aggregation and thrombosis.^13,14^ Importantly, platelet COX-1 also supports the production of other eicosanoids, including prostaglandins (PGs) such as PGE_2_, PGD_2_, PGF_2_,^15,16^ and 11- and 15- hydroxyeicosatraenoic acid (HETE),^17–19^ which have mixed actions on platelet activation, vascular function, and thrombosis.^20–27^

It has been hypothesized that the antiplatelet effect of aspirin mediated by inhibition of TxA_2_ is counterbalanced by aspirin inhibition of COX-1 and possibly COX-2 at non-platelet sites that reduces the synthesis of vasorelaxant and anti-aggregatory mediators such as PGI_2_.^28–30^ However, the complete profile of eicosanoids whose synthesis is affected by the action of aspirin on platelet COX-1 and on other tissues and how it relates to the anti- and pro-thrombotic effects of aspirin has been so far poorly characterized.

This has been difficult to appreciate because the pharmacokinetics and pharmacodynamics of aspirin are substantially different in mice and humans. Mice, in fact, require two hundred fold higher oral dose of aspirin than humans to achieve complete suppression of platelet COX-1 activity,^31,32^ but this dose of aspirin unavoidably affects COX activity in different body compartments.

While mice with global COX-1 knockout (COX-1^−/−^; hereafter, global-COX-1-ko mice) have been available for some time,^33^ selective COX-1 cell-type deletion has only very recently been documented.^34,35^

Here, we used mice with selective deletion of platelet COX-1 to mimic the selective effect of aspirin on platelet COX-1 eicosanoid synthesis. We compared this eicosanoid profile to those obtained in global-COX-1-ko mice, with lack of COX-1 activity in the entire body, and in mice treated with high-dose aspirin to achieve COX enzyme inhibition in platelet and non-platelet targets. We described that aspirin affects different networks of AA-derived eicosanoids dependent upon its action on platelet COX-1 or on non-platelet COX targets. By interrogating Ingenuity Pathway Analysis (IPA), we found that loss of COX-1-related eicosanoids in platelets is antithrombotic, but loss of COX-1- and, possibly, COX-2-related eicosanoids in the remaining of the cardiovascular system limits aspirin’s antithrombotic effects. These findings were validated by assessing platelet aggregation in vitro and thrombus formation in vivo. These results could offer a mechanistic explanation as to why the addition of aspirin to other antithrombotic drugs or increase in aspirin dose does not add antithrombotic benefit for human subjects and may increase thrombotic risk.

## 2 | MATERIALS AND METHODS

### 2.1 | Mice

We used a Cre/loxP system to create transgenic mice with deletion of COX-1 in platelets. Floxed COX-1 mice (*Ptgs1*^*flox/flox*^ mice) were generated by flanking exons 3 and 5 of the COX-1 gene with lox P sites in ES cells, which were injected into blastocysts to create chimeric mice with germline transmission. These animals have been deposited at Jackson Laboratories (USA) as strain no. 030884. COX-1^fl/fl^ mice were then crossed with *Pf4Cre* mice (provided by Prof Steve Watson, University of Birmingham, United Kingdom, and used with the permission of Prof. Radek Skoda, University of Basel, Switzerland) to specifically delete COX-1 in the megakaryocyte lineage (*Ptgs1*^*flox/flox*^;*Pf4Cre*). *Ptgs1*^*flox/flox*^;*Pf4Cre* mice were mated with *Ptgs1*^*flox/flox*^ mice to produce *Ptgs1*^*flox/flox*^;*Pf4Cre* mice (platelet-COX-1-ko mice) and *Ptgs1*^*flox/flox*^ littermate controls (hereafter control mice). This strain was maintained on a mixed C57Bl/6 and 129S4/SvJae background. Global-COX-1-ko mice on a C57BL/6 background were generated as previously described.^33^

Animals were housed with free access to food (RM1; Special Diet Services, UK) and water under a 12 hours day/night cycle. Eight- to 12-week old mice were used for all experiments and all procedures described in this study were subject to UK Home Office approval (PPL 70/8422) under “The Animals (Scientific Procedures) Act (1986) Amendment Regulations (2012)” following review by the Queen Mary University and Imperial College Animal Welfare and Ethical Review Board. Animals were randomized through allocation of sequential number at weaning (prior to genotyping) and experiments performed in this order.

### 2.2 | Complete blood counts and platelet preparation

Mice were anesthetized with intraperitoneal (i.p.) ketamine (Narketan, 100 mg/kg; Vetoquinol, UK) and xylazine (Rompun, 10 mg/kg; Bayer, Germany) and blood was collected from the inferior vena cava. Complete blood counts were performed on blood collected in EDTA (IDEXX BioResearch, Germany). To prepare platelet-rich plasma (PRP) blood was anticoagulated with sodium citrate (0.32%; Sigma, UK), diluted 1:1 in modified Tyrode’s HEPES (MTH) buffer (containing 134 mmol/L NaCl, 2.9 mmol/L KCl, 0.34 mmol/L Na_2_HPO_4_, 12 mmol/L NaHCO_3_, 1 mmol/L MgCl_2_, and 20 mmol/L HEPES, pH 7.4; all Sigma, UK) and centrifuged at 100 g for 8 minutes, followed by centrifugation of the supernatant and the buffy coat at 100 g for 6 minutes. The PRP was then used for immunofluorescence studies or to isolate platelets for immunoblot analysis. For the latter purpose, PRP was further centrifuged (750 g, 10 minutes) in the presence of PGI_2_ (PGI_2_, 1 μg/mL; Tocris Bioscience, UK) and apyrase (0.02 U/mL; Sigma, UK). The resulting platelet pellet was washed in MTH buffer containing 0.02 U/mL of apyrase (Sigma, UK) and resuspended to a final concentration of 1 × 10^9^ platelets/mL in MTH buffer.

### 2.3 | Immunoblot analysis

1 × 10^9^ platelets/mL (final) were resuspended in MTH buffer pH 7.4 containing protease inhibitor cocktail (5 mmol/L EDTA; Complete; Roche Diagnostics, Sigma, UK) and lysed by the addition of Triton X-100 (0.5% v/v). Alternatively, snap-frozen tissues were homogenized in phosphate-buffered saline (PBS) containing EDTA (10 mmol/L), Triton-X 100 (1%), phenylmethylsulfonyl fluoride (1 mmol/L), and protease inhibitor mixture (1×) using a Precellys 24 homogenizer (Bertin Instruments, France). SDS-PAGE and immunoblotting of cell lysates were performed using standard protocols. Membranes were probed with primary antibodies against mouse COX-1 (rabbit anti-mouse polyclonal IgG; Cell Signaling Technology, New England BioLabs, UK; catalogue no. 4841S; 1:1000) and GAPDH (rabbit anti-mouse monoclonal IgG; Cell Signaling Technology, New England BioLabs, UK; catalogue no. 5174S; 1:10 000) followed by secondary horseradish peroxidase-conjugated goat anti-rabbit IgG antibody (Sigma, UK; catalogue no. 12–348; 1:10 000). Proteins were detected by enhanced chemiluminescence reaction (BioRad GE Healthcare, UK) using clear-blue X-ray film or an ImageQuant-RT ECL imaging system (GE Healthcare, UK).

### 2.4 | Immunostaining and Airyscan laser scanning confocal microscopy

For platelet imaging the PRP was mixed with equal volumes of 8% paraformaldehyde in PBS and platelets were left to fix for 15 minutes at room temperature.^36^ Platelets were pelleted by centrifugation at 1000 g for 10 minutes, washed with PBS, and resuspended in PBS plus 1% BSA. To image the mixed population of platelets and leukocytes, the buffy coat was isolated from the PRP and treated with Lyse/Fix solution (BD Bioscience, Germany) to break the erythrocytes and fix the rest of the cells that were washed and resuspended in saline solution. Platelets or the mixed population of platelets and leukocytes were spotted on coverslips and incubated for 90 minutes at 37°C with saturating humidity. The coverslips were rinsed with PBS and blocked for 60 minutes with hybridization buffer (0.2% Triton X-100 in PBS plus 1% BSA and 2% donkey serum; Sigma, UK); then stained with primary antibodies against mouse α-tubulin (mouse monoclonal antibody, clone B-5-1-2; Sigma, UK, catalogue no. T5168; 1:200) and COX-1 (rabbit anti-mouse polyclonal IgG; Cell Signaling Technology, New England BioLabs, UK; catalogue no. 4841S; 1:100) diluted in the same buffer. After washing, cells were incubated for 60 minutes with Alexa 488-conjugated secondary antibody (donkey anti-mouse polyclonal IgG H+L; Life Technologies UK; catalogue no. A-21202; 1:500) and Alexa 647-conjugated secondary antibody (donkey anti-rabbit polyclonal IgG H+L; Life Technologies UK; catalogue no. A-31573; 1:500), to detect α-tubulin and COX-1, respectively, and with DAPI (25 μg/mL; Life Technologies, UK) 10 minutes, washed, postfixed, and mounted on slides with fluorescent mounting medium (Life Technologies, UK). Images were acquired with an oil immersion objective (plan-Apochromat 63X/1.4 Oil DIC M27) using an LSM 880 confocal fluorescence microscope, Axio Observer, equipped with four lasers (Diode 405–30/ Argon 458, 488, 514/DPSSS 561–10/HeNe 633), a Z-piezo stage insert and an Airyscan Detector. Immunostaining conditions, laser intensity, and exposure settings were established with minimal/undetectable levels of autofluorescence, channel cross talk, and nonspecific primary/secondary background fluorescence. Acquisition and maximum intensity projection rendering of the images was performed with Zen software (version 2.3 SP1, Zeiss, Germany). Images were exported to ImageJ (version 1.51a, NIH, USA) for final preparation. All images shown in the text are representative of at least three independent preparations.

### 2.5 | Aorta immunostaining

COX-1 expression in the aorta was assessed as previously described.^37^ Mice killed with CO_2_ were immediately perfused across the heart with PBS (20 mL) followed by 5% formalin (20 mL), and the aorta was carefully removed. The aortic tissue was then blocked (20% normal goat serum; Vector Laboratories, UK) and permeabilized (0.1% Triton X-100), before being treated with primary antibody against mouse COX-1 (rabbit anti-mouse polyclonal IgG; Cayman Chemical, USA; catalogue no. 160109; 1:50) followed by Alexa 568-conjugated goat anti-rabbit IgG secondary antibody (Life Technologies, UK; catalogue no. A-11036; 1:200). Tissues were counterstained with Alexa 488-conjugated anti-CD31 (rat anti-mouse monoclonal IgG2a,k; clone MEC13.3; Biolegend, USA; catalogue no. 102514; 1:100). After staining, aortic rings were cut open to reveal the luminal surface, mounted flat between a glass slide and coverslip with aqueous hard-set media (Vector Laboratories, UK), and pressed until the media had firmly set. The luminal surface of aortic rings was visualized with a Leica SP5 inverted confocal microscope using a 63× objective oil immersion lens. Laser and gain settings were fixed at the beginning of each imaging protocol. Nonspecific binding was excluded by subtracting the fluorescence of tissue in which the primary antibody was omitted from the staining protocol. Images were exported to Image J (version 1.51a, NIH, USA) for final preparation.

### 2.6 | Enzymatic immunoassay for 6-keto-PGF_1α_ measurement

Aortic tissue, collected as previously described^37^ <10 minutes after mouse death, was perfused with PBS and dissected into small rings (~2-mm) before being placed into individual wells of 96-well microtiter plates (Greiner Bio-one, UK) containing DMEM (200 mmol/L L-glutamine; Sigma, UK). After 30 minutes of vigorous shaking at 37°C, the media were collected and 6-keto-PGF_1α_ measured by ELISA (Enzo Life Sciences, USA).

### 2.7 | Measurement of circulating or stimulated total lipid mediators

To stimulate the release of lipid mediators in vitro, blood was treated with 50 μmol/L of A23187 (Sigma, UK) or its vehicle under stirring conditions in a light transmission aggregometer for 5 minutes. About 100 μmol/L of diclofenac sodium (Sigma, UK) and 100 U/mL of heparin (Leo Laboratories, UK) were added at the end of the activation process and plasma prepared by centrifuging the blood (12000 g, 3 minutes, 4°C). Plasma samples were stored at −80°C until lipidomic analysis.

For the analysis of the lipid mediators released in vivo, mice were anesthetized with ketamine and xylazine (i.p.) prior to intravenous (i.v.) injection with vehicle (saline) or aspirin (10 mg/kg; Flectadol, Sanofi, Italy). After 10 minutes, the synthesis of lipid mediators was stimulated by i.v. injection of arachidonic acid (AA; 2.8 mg/kg; Sigma, UK) and blood withdrawn from the inferior vena cava into lepirudin 5 minutes later. Some mice were injected with vehicle (10% EtOH, 90% saline) rather than AA for measurement of the basal circulating levels of products. Plasma samples were prepared by centrifuging the blood (12000 g, 3 minutes, 4°C) and stored at −80°C until lipidomic analysis.

### 2.8 | Liquid chromatography tandem mass spectrometry analysis of lipid mediators

Lipidomic analysis was performed by liquid chromatography tandem mass spectrometry (LC-MS/MS), as previously described.^38,39^ Briefly, 250 μL mouse plasma was spiked with 30 ng each internal standard, mixed with 1 vol of 0.1% acetic acid in 5% methanol containing 0.01 mmol/L of butylated hydroxytoluene, and extracted with 3 mL of ethyl acetate. Ethyl acetate was passed through Maestro A columns (Tecan, Mannedorf, Switzerland) under gravity flow into glass tubes containing 6 μL of 30% glycerol in methanol. Columns were washed with 1 mL of acetonitrile and samples were dried by vacuum centrifugation at 37°C, and reconstituted in 30% of ethanol. AA-derived metabolites were then separated by reverse-phase HPLC on a 1 × 150 mm, 5 μm Luna C_18_^2^ column (Phenomenex, USA) and quantified using an MDS Sciex API 3000 triple quadrupole mass spectrometer (Applied Biosystems, USA) with negative-mode electrospray ionization and multiple reaction monitoring.

### 2.9 | Platelet aggregation in whole blood

Platelet aggregation was assessed in whole blood as previously described.^40^ Briefly, half-area 96-well microtiter plates (Greiner Bio-One, UK) were precoated with hydrogenated gelatin (0.75% wt/vol; Sigma, UK) in PBS to block nonspecific activation of blood. AA (0.05–0.5 mmol/L; Sigma, UK) or its vehicle were then added to each well, followed by whole blood. The plate was then placed onto a heated plate shaker (Bioshake IQ, Q Instruments, Germany) at 37°C for 5 minutes mixing at 1200 rpm to facilitate platelet aggregation. Following mixing, 5 μL of whole blood was removed from each well and diluted 1:10 into an acid citrate dextrose solution (5 mmol/L glucose, 6.8 mmol/L trisodium citrate, 3.8 mmol/L citric acid). In some experiments, blood was preincubated with aspirin (30 μmol/L; Sigma, UK) or its vehicle for 30 minutes at 37°C. Platelets were labeled with fluorescein isothiocyanate (FITC) or allophycocyanin (APC) conjugated anti-CD41 (monoclonal rat anti-mouse, clone MWReg30; Biolegend, UK; catalogue no. 133913; 1:100) or anti- CD42d-phycoerythrin (PE) (monoclonal hamster anti-mouse/rat, clone 1C2; Biolegend, UK; catalogue no. 148503; 1:100) for 30 minutes. Samples were then diluted 1:50 in PBS containing 0.1% of formalin (Sigma, UK), 0.1% of dextrose, and 0.2% of BSA before addition of 10^4^ CountBright absolute counting beads (Thermo Fisher Scientific, UK). Labeled, diluted blood was then analyzed using a FACSCalibur flow cytometer (BD Biosciences, Germany) to determine platelet count.

### 2.10 | FeCl_3_-induced carotid artery thrombosis model

Mice were anesthetized by i.p. injection of ketamine/xylazine (100/10 mg/kg body weight). Aspirin (10 mg/kg; Flectadol; Sanofi, Italy) or saline were administered i.v. and, after 10 minutes, the left common carotid artery was exposed. A miniature Doppler flow probe (Transonic) was placed around the artery and a 5% FeCl_3_ (Sigma, UK)-soaked piece of filter paper (1 × 2 mm) was applied for 3 minutes to induce thrombus formation. Blood flow was monitored for 30 minutes and the vessel occlusion time was set as cessation of blood flow for >30 seconds.

### 2.11 | Principal component analysis and hierarchical clustering analysis

Lipid mediator abundance, expressed as ng/mL, was filtered to remove those with no signal in any sample. Data were normalized against the non-stimulated samples and the data set was uploaded to the Partek Genomics Suite (Partek, USA) for Principal Component Analysis (PCA). PCA is a multivariate analysis that provides a visual representation of the data set with score plots that show the systematic clusters among the observations (closer points represent higher similarity in the measurements). Data were also uploaded in the Partek (v6.6) software to perform hierarchical clustering using a Euclidean dissimilarity matrix.

### 2.12 | Ingenuity pathway analysis

The data set containing eicosanoids identifiers and corresponding fold changes and *P* values was uploaded onto Ingenuity Pathways Analysis (IPA) (01–13) software (QIAGEN, USA) and each eicosanoid identifier was mapped in the Ingenuity Pathways Knowledge Base (IPKB) to find cellular functions and diseases that were significantly associated with differentially synthesized eicosanoids. Fisher’s exact test was performed to calculate a *P* value determining the probability that each biological function and/or disease assigned to the data set was due to chance alone. Downstream effect analysis was used to predict changes in diseases and functions based on the direction of the change of the measured eicosanoids. In addition, networks were generated as graphical representations of the molecular relationships between eicosanoids and cellular functions.

### 2.13 | Statistical analysis

For statistical analysis, GraphPad Prism 8.0 (USA) was used to perform *t* test, one- and two-way ANOVA followed by Tukey’s test. *P* values <.05 were considered statistically significant

## 3 | RESULTS

### 3.1 | Characterization of platelet-COX-1-ko mice

**FIGURE 1:**
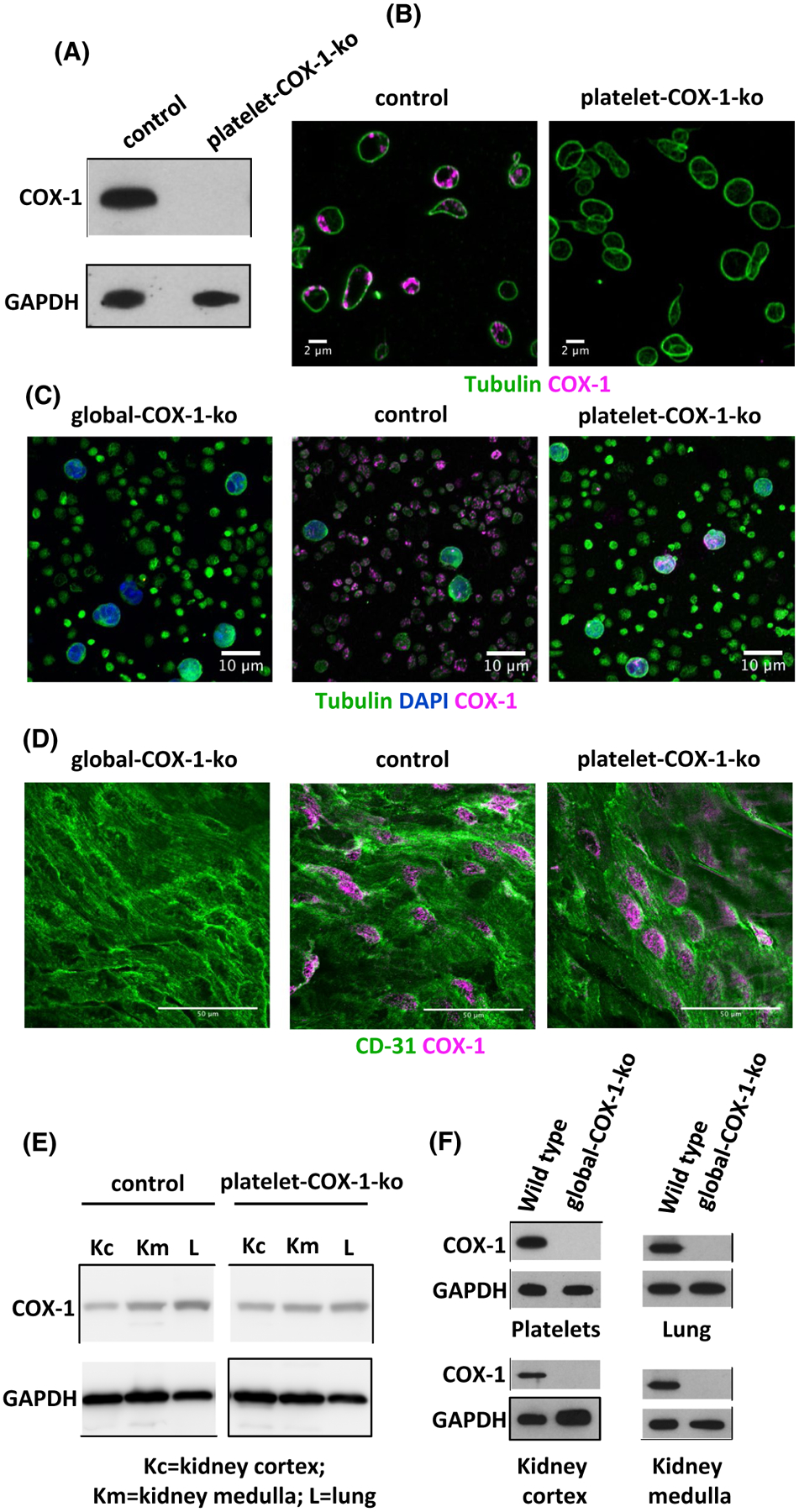
Characterization of platelet COX-1 deletion. A, Control and platelet-COX-1-ko platelet lysates were subjected to SDS-PAGE and probed for COX-1 and GAPDH as indicated. B, Fixed and permeabilized platelets from control and platelet-COX-1-ko mice were stained for tubulin (green) and COX-1 (magenta) and imaged with Airyscan laser scanning confocal microscopy. Bars represent 2 μm. C, The buffy coat was prepared from global-COX-1-ko, control, and platelet-COX-1-ko mouse blood with the mixed platelet and leukocyte populations being isolated prior to staining for tubulin (green), DAPI (blue), and COX-1 (magenta) and imaging. Bars represent 10 μm. D, Aortas from global-COX-1-ko, control, and platelet-COX-1-ko mice were fixed, permeabilized, and stained for COX-1 (magenta) and the endothelial marker CD31 (green). Aortas were then dissected into rings that were cut open to visualize their luminal surface by confocal microscopy. Bars represent 50 μm. E,F, Kidney cortex (Kc), kidney medulla (Km), lungs (L), and platelet lysates were subjected to SDS-PAGE and probed for COX-1 presence. The images are representative of at least four mice per group

Platelet-COX-1-ko mice were viable, fertile and demonstrated a normal Mendelian inheritance ratio. They had normal platelet, erythrocyte, lymphocyte, and neutrophil counts compared to their littermate controls, but slightly decreased monocyte counts (Supplemental Table 1). Western blot analysis and immunofluorescence staining confirmed the absence of COX-1 in platelets (Figure 1A,B). COX-1 was still present in leukocytes, aortic endothelial cells (Figure 1C,D) and homogenates of kidney and lungs (Figure 1E). In comparison, COX-1 was not expressed in the blood cells or in other tissues collected from global-COX-1-ko mice (Figure 1C,D,F). The effects of platelet COX-1 deletion on endothelial function were evaluated by measuring the levels of PGI_2_, the main COX-1-derived eicosanoid produced by the endothelium.^37^ We first measured the production by aortic rings of PGI_2_, determined as its breakdown product, 6-keto-PGF_1α_, and found similar levels in incubates of tissues from control (12.91 ± 2.92 ng/mL; n = 3) and platelet-COX-1-ko (8.92 ± 0.62 ng/mL; n = 3) (*P* = .66) mice, whereas levels in incubates from global-COX-1-ko mice were reduced by more than 95% (0.55 ± 0.01 ng/mL; n = 3) (*P* = .03 vs control mice). Basal circulating levels of 6-keto-PGF_1α_ in the blood tended to be higher in platelet-COX-1-ko mice (339 ± 99 pg/mL, n = 4) and control mice (214 ± 11 pg/mL, n = 4) than in global-COX-1-ko mice (60 ± 7 pg/mL, n = 4).

### 3.2 | Eicosanoid profile of in vitro-stimulated whole blood and prediction of thrombosis-related functions

**FIGURE 2:**
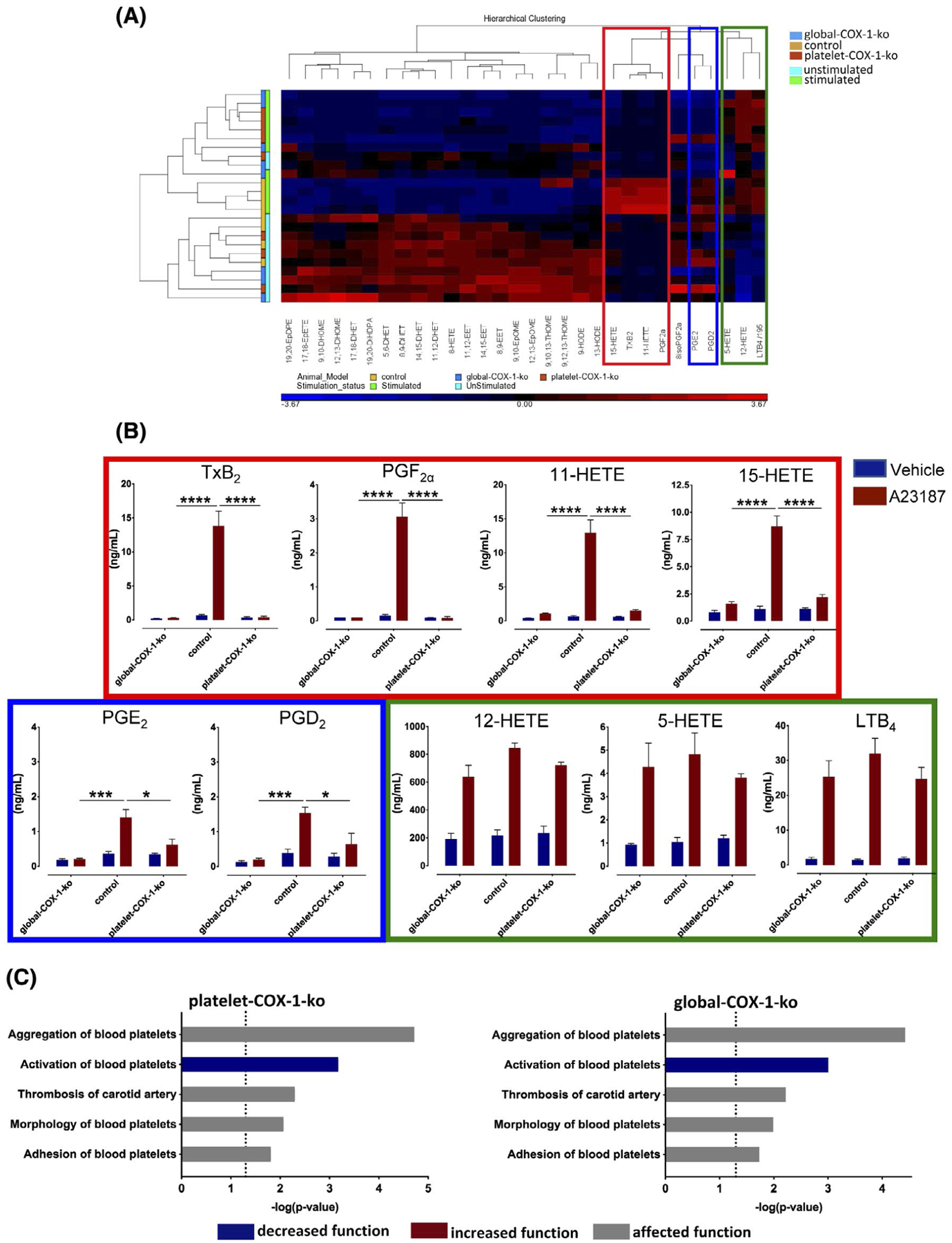
Tandem mass spectrometry analysis of lipid mediators produced in whole blood and IPA predictions of platelet activation and thrombosis. Plasma samples were prepared from mouse blood following incubation in vitro with vehicle or A23187 (50 μM) in stirring conditions (1000 rpm, 5 minutes) and subjected to tandem mass spectrometry analysis. A, Relative levels of mediators displayed in a heat map using a Z-score ranging from −4.12 (blue) to 4.12 (red). The individual products are represented on the bottom horizontal bar and their clustering on the top, while on the left the metabolites are grouped according to stimulated and unstimulated conditions and to the mouse strain. Hierarchical clustering analysis identified three different clusters of eicosanoids whose levels are significantly increased upon stimulation of whole blood with A23187 (50 μM): the cluster including TxB_2_, PGF_2α_, 11-HETE, and 15-HETE is indicated in the red box; the cluster including PGE_2_ and PGD_2_ is indicated in the blue box; and the cluster comprising 12-HETE, 5-HETE, and LTB_4_ in the green box. B, The absolute plasma levels of these eicosanoids as measured by tandem mass spectrometry. Results are from n = 4 global-COX-1-ko, control, and platelet-COX-1-ko mice for each vehicle or A23187 treatment and were analyzed by two-way ANOVA followed by Tukey’s test. Data are presented as means ± SEM. *P < .05, **P < .01, ***P < .001, and ****P < .0001. C, IPA and downstream effects analysis were used to predict the cellular events related to platelet reactivity and thrombosis affected by the increase or decrease of AA-derived eicosanoids synthesis by COX in vitro in platelet-COX-1-ko mice and global-COX-1-ko mice. In the bar charts, the disease and function categories involved in this analysis are displayed along the y-axis. The x-axis displays the −(log) significance. Longer bars are more significant than shorter bars. Functions are listed from most significant to least and the vertical dotted line denotes the cutoff for significance (*P* value of .05). Blue and grey indicates diseases or functions that are predicted as decreased or affected, respectively.

*In vitro* stimulation of whole blood with the Ca^2+^ ionophore, A23187, was used to induce receptor-independent activation of platelets and leukocytes. Thirty different released lipid mediators were detected by LC-MS/MS in blood collected from control mice, platelet-COX-1-ko mice and global-COX-1-ko mice. These data were subjected to PCA and hierarchical clustering analysis. According to the PCA analysis, lipid profiles were distinct for each mouse type; however, platelet-COX-1-ko and global-COX-1-ko mice were closely associated (data not shown). In particular, hierarchical clustering analysis of the lipid measurements identified three different clusters of AA-derived eicosanoids (Figure 2A; red, blue, and green outlined boxes), which are shown in further detail in Figure 2B (red, blue, and green outlined boxes). In the control mice, TxB_2_, the stable metabolite of TxA_2_, PGF_2α_, 11-HETE, and 15-HETE (cluster in red) were all markedly increased by incubation of whole blood with A23187. However, these increases were not observed in blood from platelet-COX-1-ko or global-COX-1-ko mice. Together, our data shows that the increases in these eicosanoids in whole blood result from the activity of platelet COX-1. The productions of PGE_2_ and PGD_2_ (cluster in blue) in response to A23187 were significantly reduced in both platelet-COX-1-ko and global-COX-1-ko mice, although not abolished, meaning that in whole blood the formation of PGE_2_ and PGD_2_ may also derive from COX-2 activity in other blood cells. Syntheses of 12-HETE, 5-HETE, and Leukotriene B_4_ (LTB_4_) (cluster in green), the first a product of 12-lipoxygenase (LOX) activity in platelets and the last two of 5-LOX activity in leukocytes, were not affected by either systemic or platelet-specific COX-1 deletion.

IPA was used to predict platelet functions and thrombosis-related processes changed on the basis of the measurements of AA COX products synthesized in vitro. Platelet activation was predicted as decreased for both platelet-COX-1-ko and global-COX-1-ko mice compared to control mice, and platelet aggregation, morphology, and adhesion and carotid artery thrombosis resulted as affected (Figure 2C). Mainly reduced TxA_2_ and PGE_2_ contributed to the eicosanoid network supporting these functional predictions (Supplemental Figure 1). These data imply that, in vitro, aspirin inhibition of COX-1 activity in platelets and in other blood cells has similar antithrombotic effects, due to the inhibition of similar eicosanoid networks.

### 3.3 | Characterization of the profiles of lipid mediators released upon administration of AA in vivo and predicted influences on thrombosis

**FIGURE 3:**
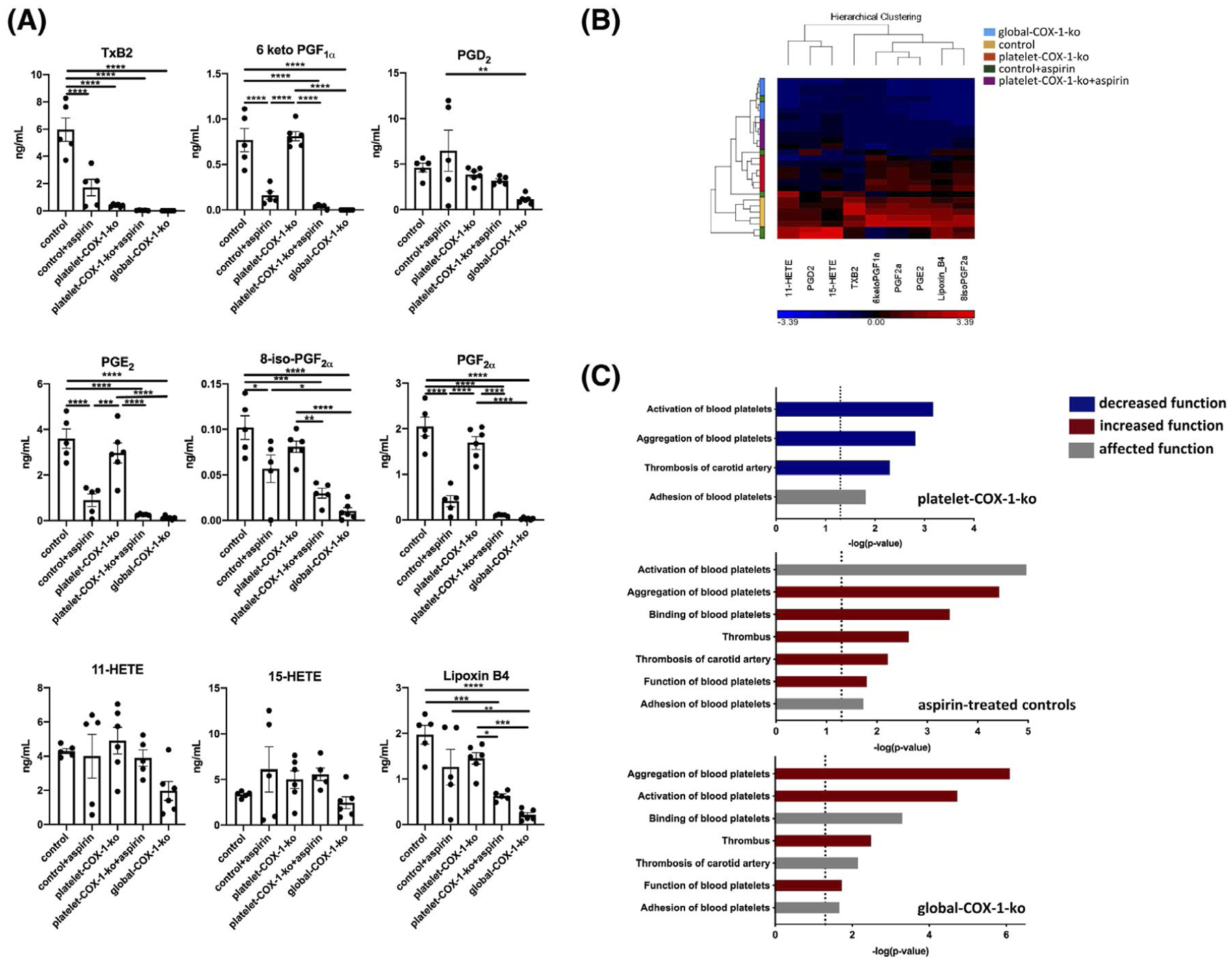
Tandem mass spectrometry analysis of eicosanoids produced in vivo and IPA predictions for platelet reactivity and thrombosis. A, Mass spectrometry measurements of COX-derived eicosanoids induced by systemic injection of AA (2.8 mg/kg, i.v.). Data are means ± SEM, n = 4–6 mice per group. *P < .05, **P < .01, ***P < .001, and ****P < .0001 (one-way ANOVA followed by Tukey’s post hoc test). B, The relative levels of eicosanoids are displayed in a heat map using a Z-score ranging from −4.20 (blue) to 4.20 (red). The individual products are identified on the bottom horizontal bar and their clustering on the top, while on the left the clustering of the mice depending on their eicosanoid signatures is shown. C, IPA was used to predict changes in processes linked to platelet reactivity and thrombosis based on the determination of in vivo eicosanoid synthesis. The bar charts show the most significant changes in platelet-COX-1-ko mice, aspirin-treated control mice, and global-COX-1-ko mice. Functions are listed from most significant to least. The x-axis displays the −(log) significance and the vertical dotted line denotes the cutoff for significance (*P* value of .05). Blue, red, and grey indicate diseases or functions that are predicted as decreased, increased, or affected, respectively.

Intravenous injection of AA (2.8 mg/kg) to control mice resulted in the generation of 36 distinct metabolites derived from AA, linoleic acid (LA), docosahexaenoic acid (DHA), and eicosapentaenoic acid (EPA) (Figure 3A provides the data for AA COX products; Supplemental Table 2 supplies the data for AA non-COX products, as well as LA, DHA, and EPA products; Supplemental Table 3 supplies the retention time and tandem MS calibrations for AA COX products). As well as inducing the production of a variety of lipid mediators, the injection of this dose of AA also reportedly induces platelet aggregation and thrombosis.^41^ Distinct profiles of AA COX eicosanoids were subsequently established for: (i) control mice; (ii) control mice treated with aspirin (10 mg/kg, i.v.); (iii) platelet-COX-1-ko mice; (iv) platelet-COX-1-ko mice treated with aspirin; and (v) global-COX-1-ko mice. These profiles were not only grouped in different clusters according to both PCA (data not shown) and hierarchical clustering analysis (Figure 3B); platelet-COX-1-ko mice (orange) clustered distinctly from control mice (yellow), but also from global-COX-1-ko mice (blue), aspirin-treated control mice (green), and aspirin-treated platelet-COX-1-ko mice (red), the last three being closely associated.

IPA of the levels of AA COX products released in vivo predicted overall protection from thrombosis for platelet-COX-1-ko mice, but not for aspirin-treated control or global-COX-1-ko mice. In particular, while the main cellular functions regulating platelet reactivity and thrombosis of the carotid artery were predicted to be reduced in platelet-COX-1-ko mice, they were predicted to be increased in both aspirin-treated control mice and global-COX-1-ko mice (Figure 3C). The eicosanoid networks associated with predicted reduction in platelet reactivity and thrombosis were dominated by dramatically reduced TxA_2_ and marginal reduction of PGE_2_ in platelet-COX-1-ko mice vs control mice. Conversely, decreased production of PGI_2_, in aspirin-treated control mice, and of PGI_2_ and PGD_2_, in global-COX-1-ko mice, underpinned the predicted increase of platelet reactivity and thrombosis (Supplemental Figure 2).

These data implied that aspirin’s inhibition of eicosanoids other than platelet-derived TxA_2_ could lead to a detrimental pro-thrombotic effect of aspirin. To further support these findings, we performed additional analyses to predict changes in vascular function that could also affect thrombosis. IPA-predicted arterial relaxation and contraction to be increased and decreased, respectively, in platelet-COX-1-ko mice; conversely vasodilation and blood flow were predicted to be decreased in aspirin-treated control mice or global-COX-1-ko mice, with a predicted increase of blood pressure and hypertension which predisposes to thrombosis (Supplemental Figure 3).

Networks of eicosanoids linked to predicted increase of vasorelaxation in platelet-COX-1-ko vs control mice similarly featured shifts in TxA_2_ and PGE_2_ and additionally marginal changes in the PGF_2α_ pathway. While reduced synthesis of PGI_2_ was linked to the predicted increase of vascular tone and blood pressure in aspirin-treated controls and global-COX-1-ko mice (Supplemental Figure 4).

### 3.4 | Validation of the functional consequences of selective blockade of platelet COX-1 versus whole body actions of aspirin

The IPA predictions of reduced platelet activation in both platelet- and global-COX-1-ko mice, based on COX-derived eicosanoid networks established upon in vitro whole blood stimulation, were validated using AA-induced platelet aggregation in whole blood. Platelet aggregation was reduced in platelets from both platelet-COX-1-ko mice (9% ± 5% aggregation, *P* = .04) and global-COX-1-ko mice (8.02 ± 8.02, *P* = .06), compared to control mice (62% ± 24% aggregation).

**FIGURE 4:**
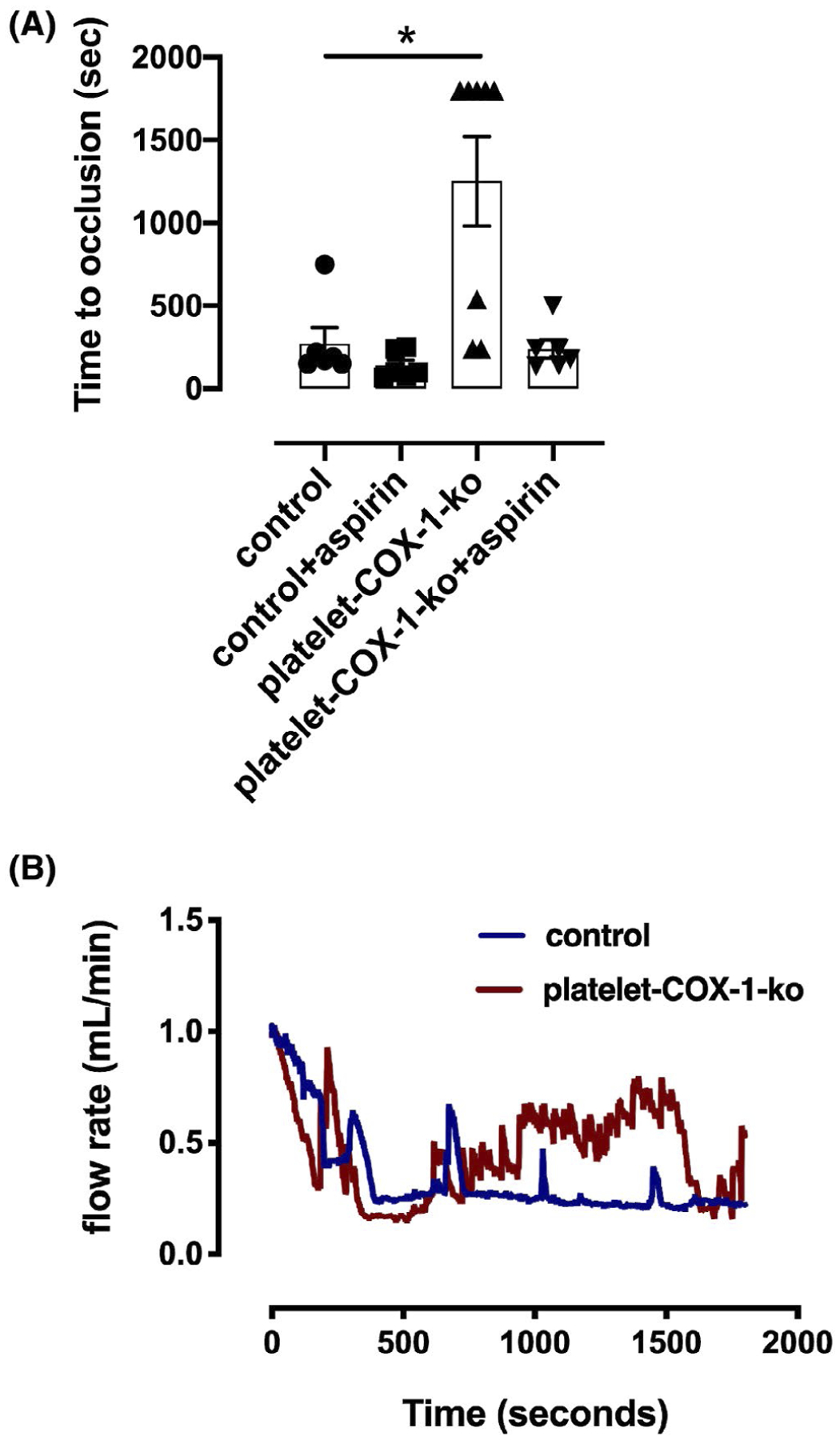
In vivo thrombosis. A, In vivo thrombus formation was studied in the carotid artery in response to exposure to 5% FeCl_3_ for 3 minutes. The time to occlusion was quantified in control mice and platelet-COX-1-ko mice under control conditions or following treatment with aspirin (10 mg/kg, i.v.). The data are means ± SEM, n = 6–8 mice per group. *P < .05 (one-way ANOVA followed by Tukey’s post hoc test). B, Traces of blood flow rate in carotid arteries during thrombosis model in (blue line) control mice and (red line) platelet-COX-1-ko mice

Thrombosis within the carotid artery was used as a model to verify the IPA predictions made on the basis of in vivo established COX-dependent eicosanoid networks. As predicted platelet-COX-1-ko mice demonstrated a significantly prolonged time for vessel occlusion compared to controls (Figure 4), that was normalized by additional treatment with aspirin (Figure 4A).

## 4 | DISCUSSION

**FIGURE 5:**
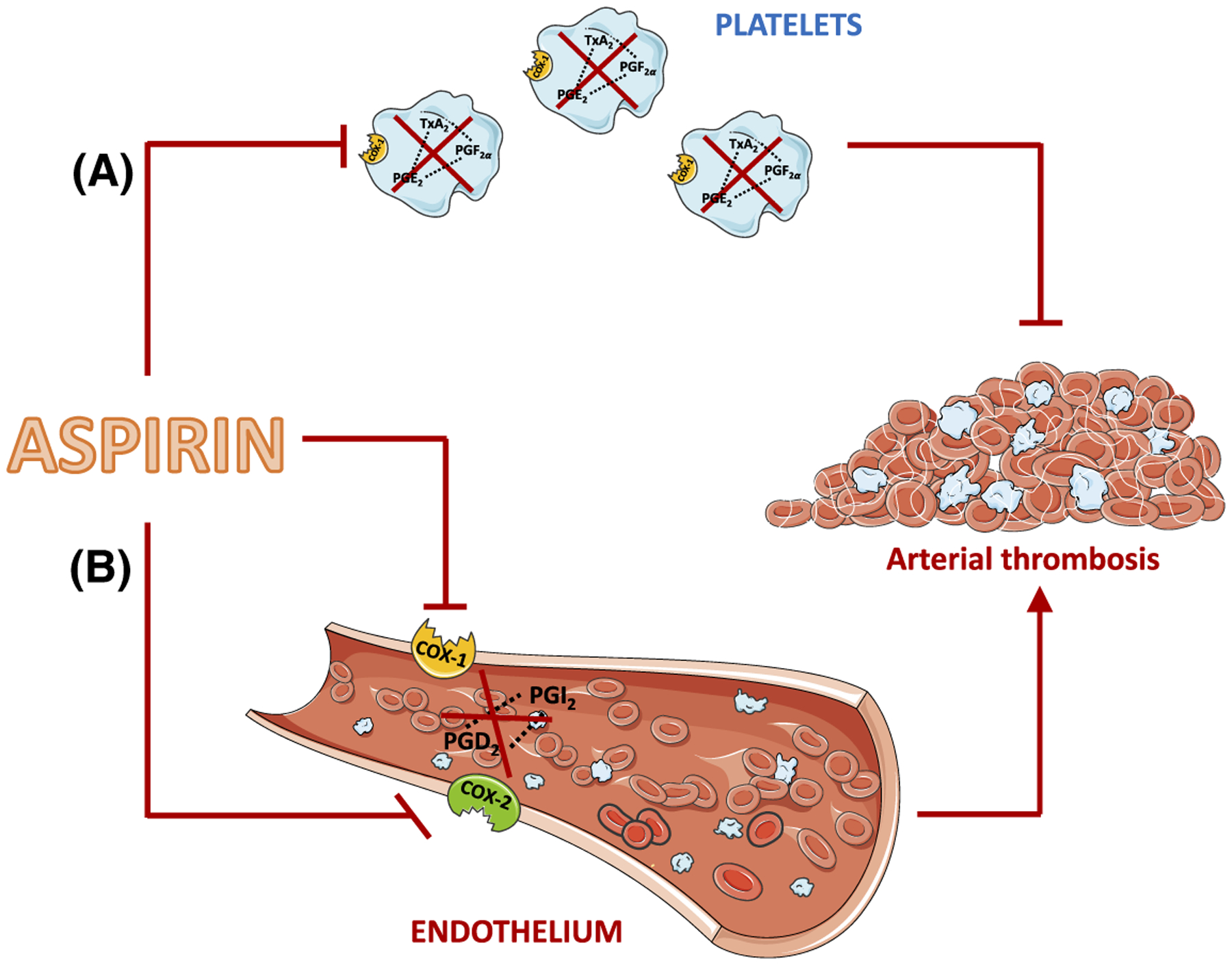
Model comparing the effects of aspirin in platelets vs the vasculature. A, Aspirin’s action in platelets, that is mimicked in p-COX-1-ko mice, results in the inhibition of COX-1-dependent pro-thrombotic eicosanoids, including TxA_2_, PGE_2_, and PGF_2α_, and in a beneficial antithrombotic effect. B, In the vasculature, aspirin blocks the activity of COX-1 and COX-2 and the synthesis of antithrombotic and vasorelaxant mediators, including PGI_2_ and PGD_2_. This effect favors thrombosis thus countering and limiting the beneficial effects of aspirin on the cardiovascular system mediated through inhibition of COX-1 in platelets. This figure was produced using Servier Medical Art (http://www.servier.com)

Numerous studies have demonstrated that prophylactic treatment with low-dose aspirin reduces the recurrence of arterial thrombotic events.^4^ However, recent reports have questioned the optimal dose of aspirin to use and the true cardiovascular benefits of aspirin in combination with other, newer therapies.^5–8,11,12^ While clinical studies have been put in place to answer these questions,^7–12^ an improved understanding of the efficacy of antithrombotic therapies that involve aspirin requires better evaluation of the mechanisms of action of aspirin on platelets and on other targets within the cardiovascular system. However, basic research studies addressing the actions of low-dose aspirin in humans provide limited information^31,42–44^ and studies using mice are hard to translate to humans because relatively very high oral aspirin doses are required to inhibit mouse platelet COX-1^32,45,46^; these elevated aspirin levels affect multiple other sites in the body. In the present work, we used platelet-COX-1-ko mice to model the specific effects of aspirin on platelet COX-1 and to define the profile of platelet-derived eicosanoids affected by aspirin. By using global-COX-1-ko mice and treating mice with aspirin we characterized the profile of eicosanoids affected by lack of COX-1 and of COX-1 and COX-2 activity, respectively, in the remainder of the body. Finally, we linked the anti- and pro-thrombotic effects of aspirin to the eicosanoid networks affected and particular platelet and non-platelet-derived eicosanoids (Figure 5).

After confirming the selective deletion of COX-1 in platelets and the maintenance of COX-1 at other important cardiovascular sites, notably the endothelium, we characterized the production of lipid mediators in whole blood in vitro and quantified thirty compounds. Deletion of platelet COX-1 was notably associated with marked reductions in the production of the eicosanoids TXA_2_, PGF_2α_, 11-HETE, and 15-HETE. These results not only support the idea that inhibition of TxA_2_ synthesis by platelets is the primary mechanism underlying the effect of aspirin in the cardiovascular system, but also suggest that additional eicosanoids dependent upon platelet COX-1 activity play roles in the net antithrombotic action of aspirin. This conclusion is consistent with our report that aspirin reduces the production of 11-HETE and 15-HETE in whole blood.^19^ We also identified for the first time PGF_2α_ as a platelet-derived PG dependent on COX-1 activity. It has been recently suggested that PGF_2α_ acts on EP and TP receptors on platelets and enhances platelet activation,^47^ which may explain some of the effects we observed. Moreover, strong evidence suggests that 15-HETE potentiates platelet aggregation, decreases PGI_2_ synthesis in endothelial cells, induces vasoconstriction and reduces the inhibitory activity of nitric oxide (NO), while stimulating endothelial and smooth muscle cell proliferation and migration.^19,48–50^ Therefore, modulation of COX-1 platelet-derived 15-HETE and PGF_2α_ may influence pathologies such as diabetes, myocardial infarction, atherosclerosis, hypertension, or cancer and their inhibition by aspirin may underpin some of the drug’s beneficial effects.^51,52^

Our data also demonstrate that the production of PGE_2_ and PGD_2_ by whole blood are partly dependent upon platelet COX-1, with the residual synthesis of these PGs in platelet-COX-1-ko mice being consistent with production by COX enzymes at other cellular sites. In vitro, PGE_2_ both inhibits and potentiates platelet aggregation, depending on its concentration and local receptor expression.^14^ Although PGD_2_ has been long regarded as a main macrophage product, Song et al have recently demonstrated that platelet activation evokes the synthesis of PGD_2_ and this response is repressed in healthy volunteers receiving low-dose aspirin.^21^ PGD_2_ interacts with platelet D-type prostanoid receptor 1 (DP1) and constrains platelet activation by increasing the activity of adenylyl cyclase.^20,21^ It also restrains thrombosis and athero-genesis in vivo,^21^ suggesting that PGD_2_ inhibition can produce a pro-thrombotic effect.

A possible limitation of our findings is that the formation of PGF_2α_, PGE_2_, and PGD_2_ in platelets might result from the nonenzymatic degradation of PGH_2_. If so in our studies, particular examination of the respective PG synthetases would be required to confirm the production of these metabolites by platelets. However, various other studies have pointed to a synthetase-dependent metabolism of PGH_2_ in platelets. For instance, blockage of TxA_2_ synthetase in human platelets is associated with an increased synthesis of PGs, including PGF_2α_, PGE_2_, and PGD_2_,^53,54^ and immunodepletion of lipocalin-like PGD synthase suppresses PGD_2_ formation in platelets stimulated with ADP.^21^

Overall the combined loss of platelet-derived COX-1 products, TxA_2_ and PGE_2_, in the platelet COX-1 mouse was associated with a reduction in platelet activation, as suggested by IPA and as we verified in platelet aggregation experiments, consistent with the widely reported antiplatelet effects of aspirin. Importantly, the productions in whole blood of the non-COX-1-dependent products, 5-HETE, 12-HETE, and LTB_4_ were unaffected by deletion of platelet COX-1 supporting the selectivity of the model.

The effects of platelet COX-1 deletion seen in blood in vitro were partially recapitulated in vivo. Notably the production of TXA_2_ was still greatly reduced. However, reductions were not seen in the levels of PGE_2_, PGF_2α_, 11-HETE, and 15-HETE indicating that in vivo platelets are at best minor contributors to the overall production of these eicosanoids. Alternatively, HETE and EET products could have been present in the AA injected to stimulate the release of eicosanoids in vivo, as a result of the autoxidation of the fatty acid (data not shown), which could explain why we did not find differences for the in vivo synthesis of 11-HETE and 15-HETE across the different mouse lines and treatments. Most notably, unlike global-COX-ko mice or control mice treated with aspirin, platelet-COX-1-ko mice had circulating levels of 6keto-PGF_1α_ that were not different from those of control mice. This result is consistent with maintenance of endothelial PGI_2_ production as demonstrated by presence of COX-1 in endothelial cells and production of PGI_2_ by isolated aortas, and confirms our finding that endothelial COX-1 is a relevant source of this potent endogenous inhibitor of platelets and thrombosis.^34,37^

The alterations in eicosanoid profile in platelet-COX-1-ko mice in vivo were associated by IPA with reduction in cardiovascular disease. In particular, the changes in the network of eicosanoids driven by reductions in platelet COX-1-dependent TxA_2_ and PGE_2_ were associated with reduced platelet activation and aggregation, and so reduced thrombosis of the carotid artery. The networks seen in aspirin-treated control mice and global-COX-1-ko mice were not associated with an antithrombotic influence, but rather with a pro-thrombotic phenotype, consistent with reduced synthesis of antithrombotic mediators PGI_2_ and PGD_2_. The networks for the aspirin-treated control mice and global-COX-1-ko mice also indicated greater drives toward vasoconstriction than predicted for platelet-COX-1 mice, consistent with suggestions that TxA_2_ and PGI_2_ have antagonistic actions on vascular tone; TXA_2_ being a potent vasoconstrictor and PGI_2_ a potent vasodilator. Notably, vasoconstriction promotes thrombosis.^55^ These findings were further confirmed by IPA predictions of reduced synthesis and accumulation of vasorelaxant mediators and signaling molecules, including cyclic adenosine mono-phosphate, NO, and cyclic guanosine monophosphate in aspirin-treated control mice (data not shown). In particular, reduced synthesis and signals of NO could be related to increased levels of asymmetric dimethylarginine (ADMA), an endogenous inhibitor of endothelial NO synthase, that could have been caused by COX-2 inhibition by aspirin. In fact, some of our group have recently reported that lack of COX-2 in mice or treatment with a COX-2 selective inhibitor leads to increased levels of ADMA in the plasma.^56,57^

Finally, to validate IPA predictions derived by the measurement of AA COX products in vivo, we compared the effects of loss of COX-1 activity in platelets and the effects of aspirin on non-platelet targets in a model of *in vivo* thrombosis. As predicted, platelet-COX-1-ko mice had an antithrombotic phenotype compared to control mice. Similarly, as predicted, addition of aspirin to platelet-COX-1-ko mice reduced the time to reach vascular occlusion indicating an increase in thrombosis. For this study, we used acute i.v. injection of a single dose of aspirin (10 mg/kg) which can reduce both COX-1 and COX-2 activity. However, we conclude that the pro-thrombotic effect of aspirin we observed is explained by inhibition of COX-1 rather than COX-2. This conclusion is supported by our recent report that acute COX-2 inhibition does not affect thrombosis but that selective deletion of COX-1 from endothelial cells is pro-thrombotic, with this latter effect overcome when COX-1 is deleted from both endothelial cells and platelets.^34^ We have previously shown that i.v. doses of aspirin lower than the one used for the present study have non-platelet effects,^58^ therefore, we did not explore the effect of escalating doses of aspirin on lipid mediator formation and thrombosis.

In conclusion, our results demonstrate that the cardiovascular actions of aspirin mediated through inhibition of platelet COX-1 or of extra-platelet aspirin targets can be explained by profiling in vitro and in vivo eicosanoid formation. These in vitro and in vivo profiles definitively show that while in vitro aspirin mimics the effect of specific platelet COX-1 inhibition, in vivo aspirin has additional non-platelet effects on eicosanoid formation that promote thrombosis. Our study suggests a mechanistic explanation for the main findings of recent trials showing no additive antithrombotic effect of aspirin when in combination with P2Y_12_ receptor antagonists.^11,12^ In agreement with previous reports,^59,60^ this study also shows the potential of combining comprehensive lipidomic analysis with platelet function analysis to assess the effectiveness of antithrombotic treatments and establish the potential for individualized antithrombotic drug regimes. The responses of an individual to aspirin are a net response resulting from the state of the platelet and the networks of eicosanoids being produced at platelet and non-platelet sites.

## Supplementary Material

Supp Fig 1

Supp Fig 2

Supp Fig 3

Supp Fig 4

Supp Table 1

Supp Table 2

Supp Table 3

## Abbreviations:

AA: arachidonic acid
ADMA: asymmetric dimethylarginine
COX: cyclooxygenase
DHA: docosahexaenoic acid
DP1: D-type prostanoid receptor 1
EP: prostaglandin E2 receptor
EPA: eicosapentaenoic acid
HETE: hydroxyeicosatraenoic acid
IPA: ingenuity pathway analysis
LA: linoleic acid
LOX: lipoxygenase
LTB4: leukotriene B4
NO: nitric oxide
PCA: principal component analysis
PG: prostaglandin
PTGS: prostaglandin G/H synthase
TP: thromboxane receptor
TX: thromboxane

## References

[1] GBD Disease and injury incidence and prevalence collaborators. Global, regional, and national incidence, prevalence, and years lived with disability for 354 diseases and injuries for 195 countries and territories, 1990–2017: a systematic analysis for the Global Burden of Disease Study 2017. Lancet (London, England). 2018;392:1789–1858. 10.1016/S0140-6736(18)32279-7 PMC6227754 30496104

[2] Jackson SP. Arterial thrombosis—insidious, unpredictable and deadly. Nat Med. 2011;17:1423–1436. 22064432 10.1038/nm.2515

[3] Ruggeri ZM. Platelets in atherothrombosis. Nat Med. 2002;8:1227–1234. 12411949 10.1038/nm1102-1227

[4] Antithrombotic Trialists’ (ATT) Collaboration. Aspirin in the primary and secondary prevention of vascular disease: collaborative meta-analysis of individual participant data from randomised trials. Lancet. 2009;373:1849–1860. 19482214 10.1016/S0140-6736(09)60503-1 PMC2715005

[5] Gargiulo G, Windecker S, Vranckx P, Gibson CM, Mehran R, Valgimigli M. A critical appraisal of aspirin in secondary prevention. Circulation. 2016;134:1881–1906. 27920074 10.1161/CIRCULATIONAHA.116.023952

[6] Welsh RC, Roe MT, Steg PG, A critical reappraisal of aspirin for secondary prevention in patients with ischemic heart disease. Am Heart J. 2016;181:92–100. 27823698 10.1016/j.ahj.2016.08.008

[7] Mahaffey KW, Wojdyla DM, Carroll K, Ticagrelor compared with clopidogrel by geographic region in the platelet inhibition and patient outcomes (PLATO) trial. Circulation. 2011;124:544–554. 21709065 10.1161/CIRCULATIONAHA.111.047498

[8] Johnston A, Jones WS, Hernandez AF. The ADAPTABLE trial and aspirin dosing in secondary prevention for patients with coronary artery disease. Curr Cardiol Rep. 2016;18:81. 27423939 10.1007/s11886-016-0749-2

[9] Vranckx P, Valgimigli M, Windecker S, Long-term ticagrelor monotherapy versus standard dual antiplatelet therapy followed by aspirin monotherapy in patients undergoing biolimus-eluting stent implantation: rationale and design of the GLOBAL LEADERS trial. EuroIntervention. 2016;12:1239–1245. 26606735 10.4244/EIJY15M11_07

[10] Baber U, Dangas G, Cohen DJ, Ticagrelor with aspirin or alone in high-risk patients after coronary intervention: rationale and design of the TWILIGHT study. Am Heart J. 2016;182:125–134. 27914492 10.1016/j.ahj.2016.09.006

[11] Mehran R, Baber U, Sharma SK, Ticagrelor with or without aspirin in high-risk patients after PCI. N Engl J Med. 2019;381:2032–2042. 31556978 10.1056/NEJMoa1908419

[12] Tomaniak M, Chichareon P, Onuma Y, Benefit and risks of aspirin in addition to ticagrelor in acute coronary syndromes. A Post Hoc analysis of the randomized GLOBAL LEADERS trial. JAMA Cardiol. 2019;4:1092–1101. 31557763 10.1001/jamacardio.2019.3355 PMC6764000

[13] Reilly M, Fitzgerald GA. Cellular activation by thromboxane A2 and other eicosanoids. Eur Heart J. 1993;14(Suppl K):88–93. 8131796

[14] Crescente M, Menke L, Chan MV, Armstrong PC, Warner TD. Eicosanoids in platelets and the effect of their modulation by aspirin in the cardiovascular system (and beyond). Br J Pharmacol. 2019;176:988–999. 29512148 10.1111/bph.14196 PMC6451075

[15] O’Brien JJ, Ray DM, Spinelli SL, The platelet as a therapeutic target for treating vascular diseases and the role of eicosanoid and synthetic PPARγ ligands. Prostaglandins Other Lipid Mediat. 2007;82:68–76. 17164134 10.1016/j.prostaglandins.2006.05.018

[16] P. Puddu AM. The complexity of platelet metabolism and its contribution to atherothrombosis. Acta Cardiol. 2009;64:157–165. 19476106 10.2143/AC.64.2.2035338

[17] Porro B, Songia P, Squellerio I, Tremoli E, Cavalca V. Analysis, physiological and clinical significance of 12-HETE: a neglected platelet-derived 12-lipoxygenase product. J Chromatogr B. 2014;964:26–40. 10.1016/j.jchromb.2014.03.015 24685839

[18] Kirkby NS, Reed DM, Edin ML, Inherited human group IVA cytosolic phospholipase A 2 deficiency abolishes platelet, endothelial, and leucocyte eicosanoid generation. FASEB J. 2015;29:4568–4578. 26183771 10.1096/fj.15-275065 PMC4608906

[19] Rauzi F, Kirkby NS, Edin ML, Aspirin inhibits the production of proangiogenic 15(S)-HETE by platelet cyclooxygenase-1. FASEB J. 2016;30:4256–4266. 27633788 10.1096/fj.201600530R PMC5102123

[20] Braun M, Schrör K. Prostaglandin D2 relaxes bovine coronary arteries by endothelium-dependent nitric oxide-mediated cGMP formation. Circ Res. 1992;71:1305–1313. 1385004 10.1161/01.res.71.6.1305

[21] Song W-L, Stubbe J, Ricciotti E, Niacin and biosynthesis of PGD(2) by platelet COX-1 in mice and humans. J Clin Invest. 2012;122:1459–1468. 22406532 10.1172/JCI59262 PMC3314457

[22] Gross S, Tilly P, Hentsch D, Vonesch J-L, Fabre J-E. Vascular wall–produced prostaglandin E2 exacerbates arterial thrombosis and atherothrombosis through platelet EP3 receptors. J Exp Med. 2007;204:311–320. 17242161 10.1084/jem.20061617 PMC2118736

[23] Petrucci G, De Cristofaro R, Rutella S, Prostaglandin E2 differentially modulates human platelet function through the prostanoid EP2 and EP3 receptors. J Pharmacol Exp Ther. 2011;336:391–402. 21059804 10.1124/jpet.110.174821

[24] Glenn JR, White AE, Iyu D, Heptinstall S. PGE(2) reverses G(s)-mediated inhibition of platelet aggregation by interaction with EP3 receptors, but adds to non-G(s)-mediated inhibition of platelet aggregation by interaction with EP4 receptors. Platelets. 2012;23:344–351. 22436052 10.3109/09537104.2011.625575

[25] Croset M, Sala A, Folco G, Lagarde M. Inhibition by lipoxygenase products of TXA2-like responses of platelets and vascular smooth muscle. Biochem Pharmacol. 1988;37:1275–1280. 2965590 10.1016/0006-2952(88)90782-4

[26] Johnson EN, Brass LF, Funk CD. Increased platelet sensitivity to ADP in mice lacking platelet-type 12-lipoxygenase. Proc Natl Acad Sci. 1998;95:3100–3105. 9501222 10.1073/pnas.95.6.3100 PMC19701

[27] Maskrey BH, Rushworth GF, Law MH, 12-hydroxyeicosatetraenoic acid is associated with variability in aspirin-induced platelet inhibition. J Inflamm (Lond). 2014;11:33. 25349537 10.1186/s12950-014-0033-4 PMC4209229

[28] FitzGerald GA, Oates JA, Hawiger J, Endogenous biosynthesis of prostacyclin and thromboxane and platelet function during chronic administration of aspirin in man. J Clin Invest. 1983;71:676–688. 6338043 10.1172/JCI110814 PMC436917

[29] Leadbeater PDM, Kirkby NS, Thomas S, Aspirin has little additional anti-platelet effect in healthy volunteers receiving prasugrel. J Thromb Haemost. 2011;9:2050–2056. 21794076 10.1111/j.1538-7836.2011.04450.x PMC3338354

[30] Warner TD, Armstrong PCJ, Curzen NP, Mitchell JA. Dual anti-platelet therapy in cardiovascular disease: does aspirin increase clinical risk in the presence of potent P2Y12 receptor antagonists? Heart. 2010;96:1693–1694. 20956485 10.1136/hrt.2010.205724

[31] Patrignani P, Filabozzi P, Patrono C. Selective cumulative inhibition of platelet thromboxane production by low-dose aspirin in healthy subjects. J Clin Invest. 1982;69:1366–1372. 7045161 10.1172/JCI110576 PMC370209

[32] Armstrong PC, Kirkby NS, Zain ZN, Emerson M, Mitchell JA, Warner TD. Thrombosis is reduced by inhibition of COX-1, but unaffected by inhibition of COX-2, in an acute model of platelet activation in the mouse. PLoS ONE. 2011;6:e20062. 21629780 10.1371/journal.pone.0020062 PMC3100333

[33] Langenbach R, Morham SG, Tiano HF, Prostaglandin synthase 1 gene disruption in mice reduces arachidonic acid-induced inflammation and indomethacin-induced gastric ulceration. Cell. 1995;83:483–492. 8521478 10.1016/0092-8674(95)90126-4

[34] Mitchell JA, Shala F, Elghazouli Y, Cell-specific gene deletion reveals the antithrombotic function of COX1 and explains the vascular COX1/prostacyclin paradox. Circ Res. 2019;125:847–854. 31510878 10.1161/CIRCRESAHA.119.314927 PMC6791564

[35] Sacco A, Bruno A, Contursi A, Platelet-specific deletion of Cyclooxygenase-1 ameliorates dextran sulfate sodium-induced colitis in mice. J Pharmacol Exp Ther. 2019;370:416–426. 31248980 10.1124/jpet.119.259382

[36] Kahr WHA, Lo RW, Li L, Abnormal megakaryocyte development and platelet function in Nbeal2^(−/−)^ mice. Blood. 2013;122:3349–3358. 23861251 10.1182/blood-2013-04-499491 PMC3953091

[37] Kirkby NS, Lundberg MH, Harrington LS, Cyclooxygenase-1, not cyclooxygenase-2, is responsible for physiological production of prostacyclin in the cardiovascular system. Proc Natl Acad Sci. 2012;109:17597–17602. 23045674 10.1073/pnas.1209192109 PMC3491520

[38] Newman JW, Watanabe T, Hammock BD. The simultaneous quantification of cytochrome P450 dependent linoleate and arachidonate metabolites in urine by HPLC-MS/MS. J Lipid Res. 2002;43:1563–1578. 12235189 10.1194/jlr.d200018-jlr200

[39] Edin ML, Hamedani BG, Gruzdev A, Epoxide hydrolase 1 (EPHX1) hydrolyzes epoxyeicosanoids and impairs cardiac recovery after ischemia. J Biol Chem. 2018;293:3281–3292. 29298899 10.1074/jbc.RA117.000298 PMC5836130

[40] Armstrong PCJ, Kirkby NS, Chan MV, Novel whole blood assay for phenotyping platelet reactivity in mice identifies ICAM-1 as a mediator of platelet-monocyte interaction. Blood. 2015;126:e11–e18. 26215112 10.1182/blood-2015-01-621656 PMC4559940

[41] Griffett EM, Kinnon SM, Kumar A, Lecker D, Smith GM, Tomich EG. Effects of 6-[p-(4-phenylacetylpiperazin-1-yl)phenyl]-4,5-di-hydro-3(2H)pyridazinone (CCI 17810) and aspirin on platelet aggregation and adhesiveness. Br J Pharmacol. 1981;72:697–705. 7284687 10.1111/j.1476-5381.1981.tb09151.x PMC2071647

[42] Vane JR. Inhibition of prostaglandin synthesis as a mechanism of action for aspirin-like drugs. Nat New Biol. 1971;231:232–235. 5284360 10.1038/newbio231232a0

[43] Cerletti C, Livio M, De Gaetano G. Non-steroidal anti-inflammatory drugs react with two sites on platelet cyclo-oxygenase. Evidence from “in vivo” drug interaction studies in rats. Biochim Biophys Acta. 1982;714:122–128. 6799004 10.1016/0304-4165(82)90133-7

[44] Patrono C, García Rodríguez LA, Landolfi R, Baigent C. Low-dose aspirin for the prevention of atherothrombosis. N Engl J Med. 2005;353:2373–2383. 16319386 10.1056/NEJMra052717

[45] DiMinno G, Silver MJ. Mouse antithrombotic assay: a simple method for the evaluation of antithrombotic agents in vivo. Potentiation of antithrombotic activity by ethyl alcohol. J Pharmacol Exp Ther. 1983;225:57–60. 6834277

[46] Shen ZQ, Liang Y, Chen ZH, Liu WP, Duan L. Effects of copper-aspirin complex on platelet aggregation and thrombosis in rabbits and mice. J Pharm Pharmacol. 1998;50:1275–1279. 9877314 10.1111/j.2042-7158.1998.tb03345.x

[47] Kashiwagi H, Yuhki K, Imamichi Y, Prostaglandin F2α facilitates platelet activation by acting on prostaglandin E2 receptor subtype EP3 and thromboxane A2 receptor TP in mice. Thromb Haemost. 2019;119:1311–1320. 31129913 10.1055/s-0039-1688906

[48] Mayer B, Moser R, Gleispach H, Kukovetz WR. Possible inhibitory function of endogenous 15-hydroperoxyeicosatetraenoic acid on prostacyclin formation in bovine aortic endothelial cells. Biochim Biophys Acta–Lipids Lipid Metab. 1986;875:641–653. 10.1016/0005-2760(86)90088-3 3081039

[49] Setty BN, Stuart MJ. 15-Hydroxy-5,8,11,13-eicosatetraenoic acid inhibits human vascular cyclooxygenase. Potential role in diabetic vascular disease. J Clin Invest. 1986;77:202–211. 3080473 10.1172/JCI112277 PMC423328

[50] Setty BN, Werner MH, Hannun YA, Stuart MJ. 15-Hydroxyeicosatetraenoic acid-mediated potentiation of thrombin-induced platelet functions occurs via enhanced production of phosphoinositide-derived second messengers–sn-1,2-diacylglycerol and inositol-1,4,5-trisphosphate. Blood. 1992;80:2765–2773. 1333301

[51] Berg K, Jynge P, Bjerve K, Skarra S, Basu S, Wiseth R. Oxidative stress and inflammatory response during and following coronary interventions for acute myocardial infarction. Free Radic Res. 2005;39:629–636. 16036341 10.1080/10715760400028027

[52] Zhang J, Gong Y, Yu Y. PG F(2α) receptor: a promising therapeutic target for cardiovascular disease. Front Pharmacol. 2010;1:116. 21607067 10.3389/fphar.2010.00116 PMC3095374

[53] Uotila P, Matintalo M. Inhibition of thromboxane synthetase by OKY-1581 stimulates the formation of PGE2, PGF2α, PGD2 AND 6-KETO-PGF1α in human platelets. Prostaglandins Leukot Med. 1984;14:41–46. 6427791 10.1016/0262-1746(84)90022-2

[54] McAuliffe SJG, Moors JA, Snow HM, Wayne M, Jessup R. Redirection of arachidonic acid metabolism by ICI D1542: effects on thrombus formation in the coronary artery of the anaesthetized dog. Br J Pharmacol. 1993;108:901–906. 8485629 10.1111/j.1476-5381.1993.tb13484.x PMC1908127

[55] Berry JD, Lloyd-Jones DM, Garside DB, Greenland P. Framingham risk score and prediction of coronary heart disease death in young men. Am Heart J. 2007;154:80–86. 17584558 10.1016/j.ahj.2007.03.042 PMC2279177

[56] Ahmetaj-Shala B, Kirkby NS, Knowles R, Evidence that links loss of cyclooxygenase-2 with increased asymmetric dimethylarginine. Circulation. 2015;131:633–642. 25492024 10.1161/CIRCULATIONAHA.114.011591 PMC4768634

[57] Kirkby NS, Raouf J, Ahmetaj-Shala B, Mechanistic definition of the cardiovascular mPGES-1/COX-2/ADMA axis. Cardiovasc Res. 2020. 10.1093/cvr/cvz290 PMC7519887 31688905

[58] Kirkby NS, Chan MV, Lundberg MH, Aspirin-triggered 15-epi-lipoxin A4 predicts cyclooxygenase-2 in the lungs of LPS-treated mice but not in the circulation: implications for a clinical test. FASEB J. 2013;27:3938–3946. 23792301 10.1096/fj.12-215533 PMC3973905

[59] Peng B, Geue S, Coman C, Identification of key lipids critical for platelet activation by comprehensive analysis of the platelet lipidome. Blood. 2018;132:e1–e12. 29784642 10.1182/blood-2017-12-822890 PMC6137561

[60] McFadyen JD, Peter K. Platelet lipidomics and function: joining the dots. Blood. 2018;132:465–466. 30072414 10.1182/blood-2018-06-854950

