# Supplementary material for: Profiling the eicosanoid networks that underlie the anti- and pro-thrombotic effects of aspirin
Source: FASEB J. Author manuscript; Available in PMC 2022 Aug 8. (PMC9359103; doi:10.1096/fj.202000312R)
Supplement: Supp Fig 1 [file NIHMS1825952-supplement-Supp_Fig_1.pptx]

## Slide 1
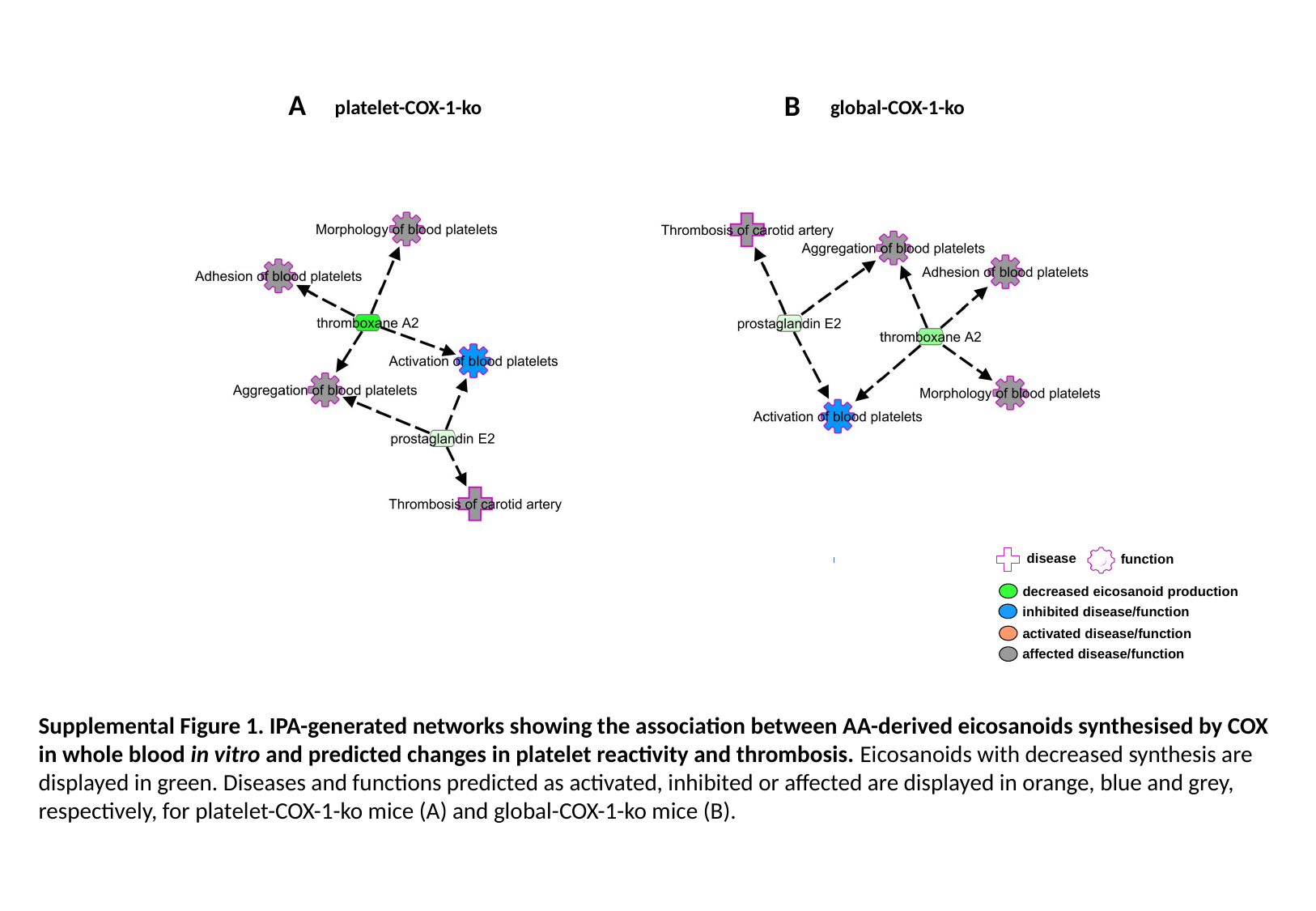

A
B
platelet-COX-1-ko
global-COX-1-ko
disease
function
decreased eicosanoid production
inhibited disease/function
activated disease/function
affected disease/function
Supplemental Figure 1. IPA-generated networks showing the association between AA-derived eicosanoids synthesised by COX in whole blood in vitro and predicted changes in platelet reactivity and thrombosis. Eicosanoids with decreased synthesis are displayed in green. Diseases and functions predicted as activated, inhibited or affected are displayed in orange, blue and grey, respectively, for platelet-COX-1-ko mice (A) and global-COX-1-ko mice (B).
