# Supplementary material for: Profiling the eicosanoid networks that underlie the anti- and pro-thrombotic effects of aspirin
Source: FASEB J. Author manuscript; Available in PMC 2022 Aug 8. (PMC9359103; doi:10.1096/fj.202000312R)
Supplement: Supp Fig 2 [file NIHMS1825952-supplement-Supp_Fig_2.pptx]

## Slide 1
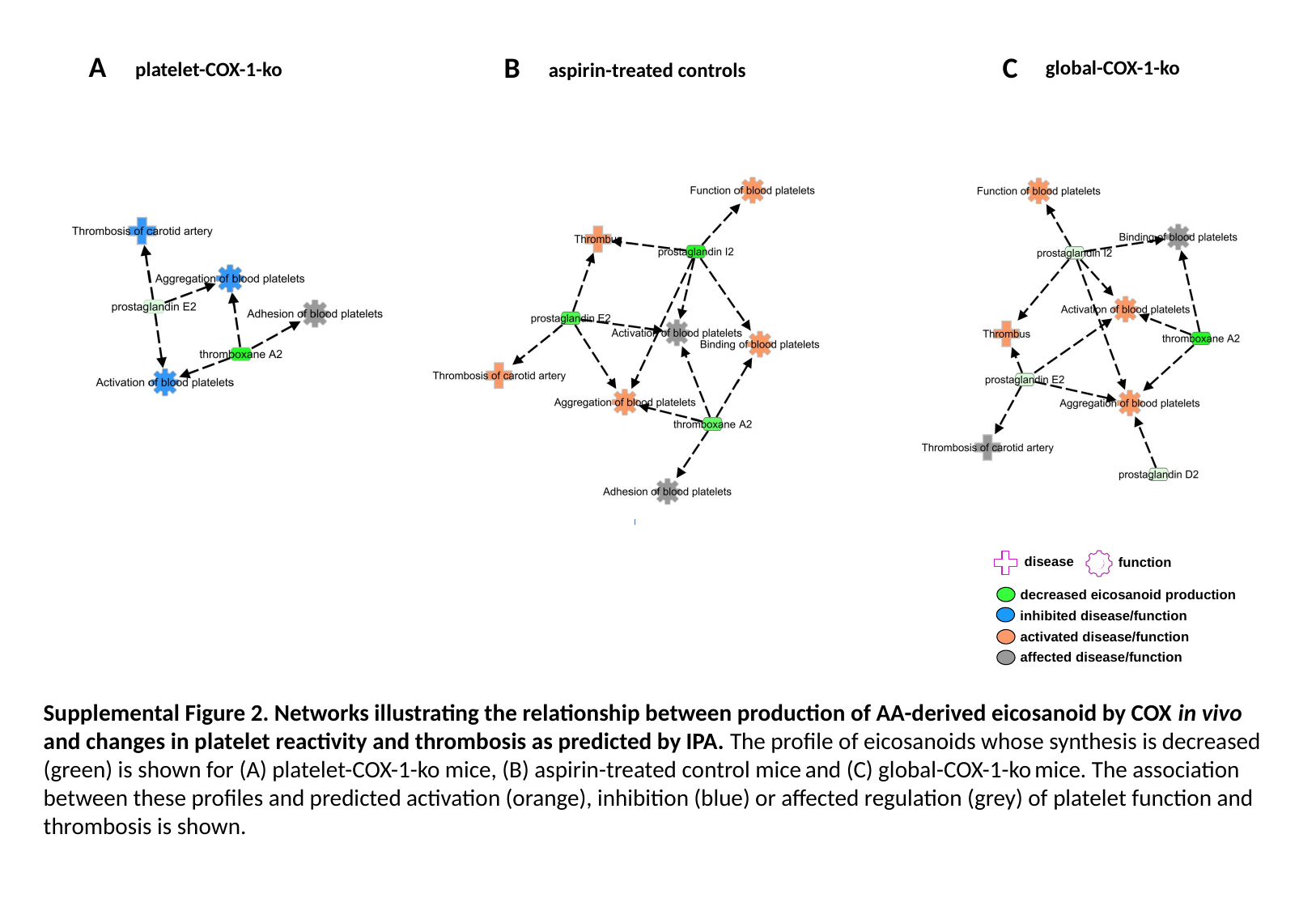

A
B
C
global-COX-1-ko
platelet-COX-1-ko
aspirin-treated controls
disease
function
decreased eicosanoid production
inhibited disease/function
activated disease/function
affected disease/function
Supplemental Figure 2. Networks illustrating the relationship between production of AA-derived eicosanoid by COX in vivo and changes in platelet reactivity and thrombosis as predicted by IPA. The profile of eicosanoids whose synthesis is decreased (green) is shown for (A) platelet-COX-1-ko mice, (B) aspirin-treated control mice and (C) global-COX-1-ko mice. The association between these profiles and predicted activation (orange), inhibition (blue) or affected regulation (grey) of platelet function and thrombosis is shown.
