# Supplementary material for: Profiling the eicosanoid networks that underlie the anti- and pro-thrombotic effects of aspirin
Source: FASEB J. Author manuscript; Available in PMC 2022 Aug 8. (PMC9359103; doi:10.1096/fj.202000312R)
Supplement: Supp Fig 3 [file NIHMS1825952-supplement-Supp_Fig_3.pptx]

## Slide 1
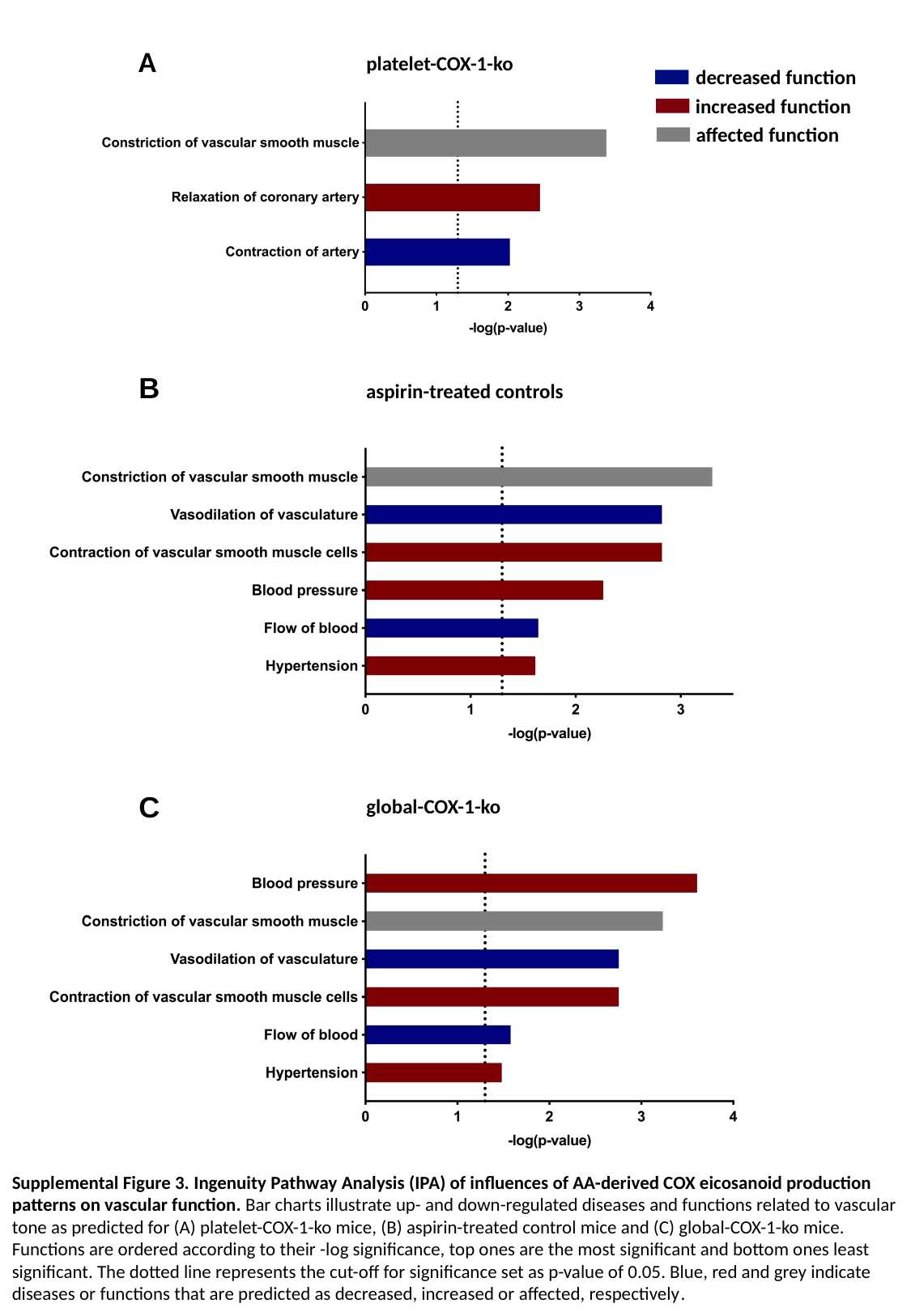

A
platelet-COX-1-ko
decreased function
increased function
affected function
B
aspirin-treated controls
C
global-COX-1-ko
Supplemental Figure 3. Ingenuity Pathway Analysis (IPA) of influences of AA-derived COX eicosanoid production patterns on vascular function. Bar charts illustrate up- and down-regulated diseases and functions related to vascular tone as predicted for (A) platelet-COX-1-ko mice, (B) aspirin-treated control mice and (C) global-COX-1-ko mice. Functions are ordered according to their -log significance, top ones are the most significant and bottom ones least significant. The dotted line represents the cut-off for significance set as p-value of 0.05. Blue, red and grey indicate diseases or functions that are predicted as decreased, increased or affected, respectively.
