# Supplementary material for: Profiling the eicosanoid networks that underlie the anti- and pro-thrombotic effects of aspirin
Source: FASEB J. Author manuscript; Available in PMC 2022 Aug 8. (PMC9359103; doi:10.1096/fj.202000312R)
Supplement: Supp Fig 4 [file NIHMS1825952-supplement-Supp_Fig_4.pptx]

## Slide 1
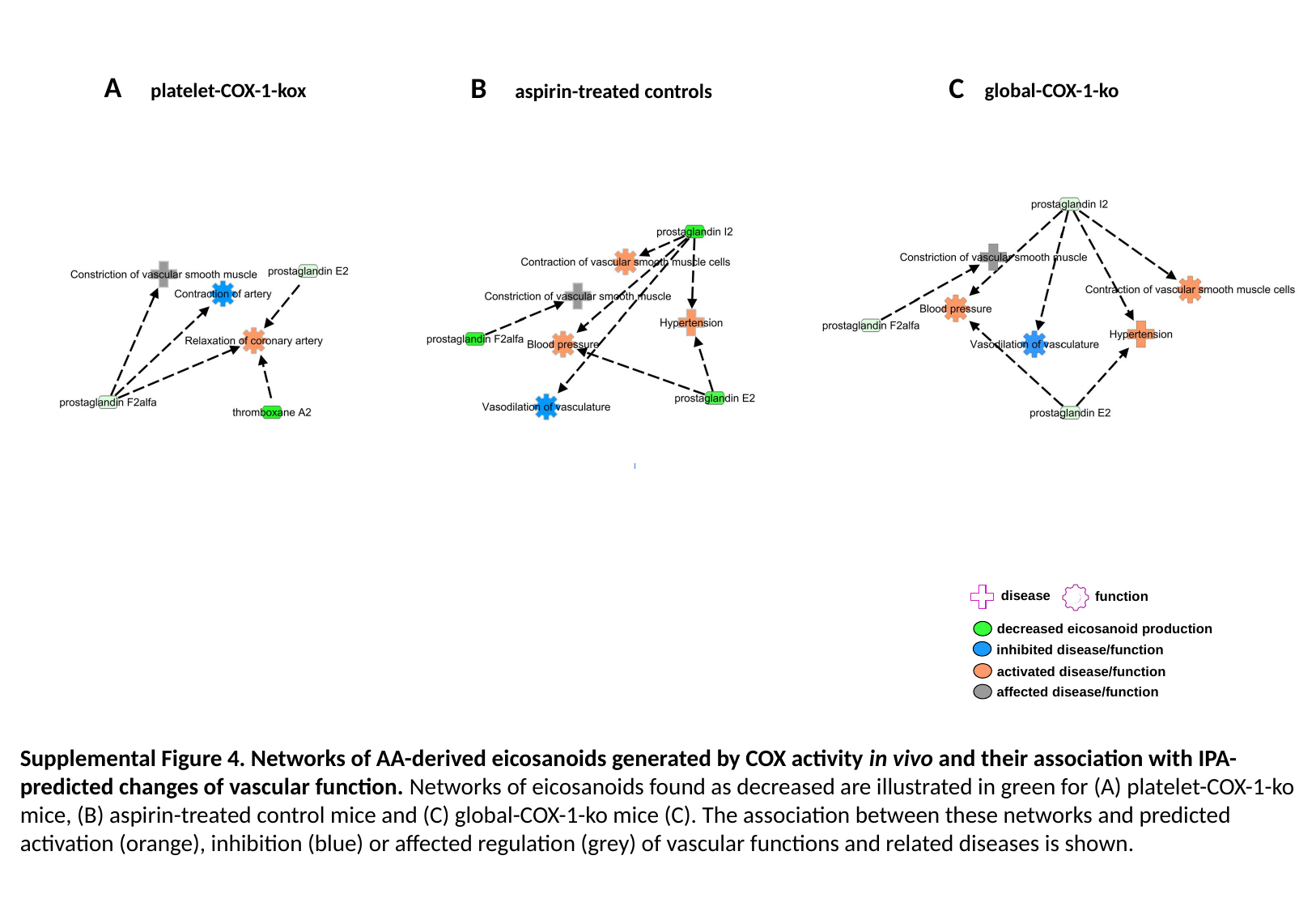

A
B
C
platelet-COX-1-kox
global-COX-1-ko
aspirin-treated controls
disease
function
decreased eicosanoid production
inhibited disease/function
activated disease/function
affected disease/function
Supplemental Figure 4. Networks of AA-derived eicosanoids generated by COX activity in vivo and their association with IPA-predicted changes of vascular function. Networks of eicosanoids found as decreased are illustrated in green for (A) platelet-COX-1-ko mice, (B) aspirin-treated control mice and (C) global-COX-1-ko mice (C). The association between these networks and predicted activation (orange), inhibition (blue) or affected regulation (grey) of vascular functions and related diseases is shown.
