# Supplementary material for: Profiling the eicosanoid networks that underlie the anti- and pro-thrombotic effects of aspirin
Source: FASEB J. Author manuscript; Available in PMC 2022 Aug 8. (PMC9359103; doi:10.1096/fj.202000312R)
Supplement: Supp Table 1 [file NIHMS1825952-supplement-Supp_Table_1.docx]

|  | *control* | *platelet-COX-1 ko* | *P* |
| --- | --- | --- | --- |
| **Erythrocytes (T/L)** | 9.9±0.3 | 9.98±0.3 | NS |
| **Haemoglobin (g/dL)** | 14.4±0.4 | 15.23±0.5 | NS |
| **PCV (%)** | 56.6±1.1 | 54.63±2.6 | NS |
| **MCV (fL)** | 57.1±0.4 | 54.63±1.7 | NS |
| **Platelets (G/L)** | 522±108 | 495±106 | NS |
| **Lymphocytes (absolute) (/μL)** | 7300±895 | 5890±859 | NS |
| **Neutrophiles (absolute) (/μL)** | 1038±195 | 704±135 | NS |
| **Monocytes (absolute) (/μL)** | 279±39 | 156±44 | .028 |

**Table S1. Complete blood counts in control and platelet-COX-1-ko mice**

Results are expressed as mean±SEM (n=8 in each group). PCV, packed cell volume; MCV, mean corpuscular volume; NS, no significant differences between groups.
