# Supplementary material for: Profiling the eicosanoid networks that underlie the anti- and pro-thrombotic effects of aspirin
Source: FASEB J. Author manuscript; Available in PMC 2022 Aug 8. (PMC9359103; doi:10.1096/fj.202000312R)
Supplement: Supp Table 2 [file NIHMS1825952-supplement-Supp_Table_2.docx]

|  | **control** | **control+aspirin** | **platelet-COX-1-ko** | **platelet-COX-1-ko+aspirin** | **global-COX-1-ko** |
| --- | --- | --- | --- | --- | --- |
| **5-HETE** | 213.862±16.358 | 271.336±26.468 | 319.598±57.551 | 273.834±14.021 | 239.516±40.075 |
| **8-HETE** | 220.572±15.672 | 282.066±27.714 | 327.814±59.009 | 279.257±14.280 | 251.950±42.295 |
| **12-HETE** | 1356.153±95.468 | 1739.243±166.066 | 2043.025±369.575 | 1729.632±96.574 | 1544.734±266.885 |
| **19-HETE** | 0.251±0.059 | 0.747±0.121 | 0.173±0.027 | 0.157±0.053 | 0.141±0.029 |
| **8,9-EET** | 0.197±0.107 | 1.440±0.432 | 0.777±0.111 | 1.04±0.210 | 0.769±0.242 |
| **14,15-EET** | 0.161±0.037 | 1.174±0.538 | 0.223±0.01 | 0.461±0.092 | 0.289±0.033 |
| **5,6-DHET** | 0.399±0.037 | 0.572±0.049 | 0.642±0.098 | 0.531±0.042 | 0.407±0.059 |
| **8,9-DHET** | 1.432±0.136 | 1.640±0.201 | 1.866±0.224 | 1.343±0.07 | 1.423±0.131 |
| **11,12-DHET** | 1.999±0.182 | 3.953±0.246 | 3.518±0.503 | 2.929±0.207 | 2.453±0.447 |
| **14,15-DHET** | 2.405±0.266 | 5.391±0.353 | 4.132±0.528 | 3.501±0.243 | 2.691±0.367 |
| **Lipoxin A4** | 0.496±0.014 | 6.612±3.931 | 0.723±0.098 | 1.045±0.053 | 0.454±0.053 |
| **9-HODE** | 3.081±0.638 | 6.477±1.483 | 11.793±2.312 | 5.329±0.78 | 9.745±3.201 |
| **13-HODE** | 19.044±3.734 | 32.099±12.928 | 49.707±10.286 | 19.538±2.875 | 37.674±9.397 |
| **9,10-DHOME** | 4.68±0.899 | 5.586±1.836 | 3.904±0.691 | 2.632±0.303 | 4.144±0.288 |
| **12,13-DHOME** | 13.831±2.694 | 17.186±5.233 | 11.481±2.062 | 7.187±0.864 | 13.556±1.086 |
| **9,10-EpOME** | 4.470±0.681 | 4.702±1.533 | 3.428±0.583 | 3.762±1.05 | 3.996±0.398 |
| **12,13- EpOME** | 4.105±0.617 | 3.899±1.407 | 2.595±0.398 | 3.322±0.997 | 3.319±0.552 |
| **7,8-DiHDPA** | 0.486±0.048 | 0.432±0.087 | 0.319±0.031 | 0.997±0.029 | 0.464±0.045 |
| **13,14-DiHDPA** | 0.287±0.050 | 0.569±0.114 | 0.259±0.034 | 0.23±0.028 | 0.273±0.015 |
| **16,17-DiHDPA** | 0.639±0.119 | 1.539±0.324 | 0.599±0.076 | 0.481±0.058 | 0.624±0.041 |
| **19,20-DiHDPA** | 2.6±0.401 | 4.587±0.695 | 1.751±0.18 | 1.546±0.228 | 2.071±0.195 |
| **7,8-EpDPA** | 58.739±6.318 | 60.835±3.991 | 78.215±8.029 | 69.039±6.388 | 117.179±9.395 |
| **10,11-EpDPA** | 0.138±0.0176 | 0.079±0.008 | 0.152±0.01 | 0.107±0.015 | 0.287±0.032 |
| **16,17-EpDPA** | 0.116±0.025 | 0.089±0.026 | 0.157±0.0157 | 0.129±0.029 | 0.336±0.052 |
| **19,20-EpDPE** | 2.366±0.346 | 2.865±0.428 | 3.741±0.380 | 2.959±0.448 | 4.545±0.320 |
| **17,18-DiHETE** | 4.889±1.149 | 6.099±1.166 | 4.622±0.718 | 3.545±0.636 | 3.497±0.290 |
| **17,18-EpETE** | 0.643±0.085 | 0.411±0.079 | 0.605±0.055 | 0.433±0.0377 | 0.968±0.103 |

**Table S2. Metabolites measured in whole blood after *in vivo* stimulation with AA**. The data are reported as means ± SEM, n=5-6.

**EPA METABOLITES**

**(ng/mL)**

**DHA METABOLITES**

**(ng/mL)**

**AA**

**NON-COX METABOLITES**

**(ng/mL)**

**LA METABOLITES**

**(ng/mL)**
