# Supplementary material for: Profiling the eicosanoid networks that underlie the anti- and pro-thrombotic effects of aspirin
Source: FASEB J. Author manuscript; Available in PMC 2022 Aug 8. (PMC9359103; doi:10.1096/fj.202000312R)
Supplement: Supp Table 3 [file NIHMS1825952-supplement-Supp_Table_3.docx]

| **compound** | **Q1** | **Q3** | **Retention time** |
| --- | --- | --- | --- |
|  |  |  |  |
| **6ketoPGF_1α_** | 369 | 163 | 1 |
| **TXB_2_** | 369 | 169 | 1.8 |
| **8isoPGF_2α_** | 353 | 193 | 1.8 |
| **PGF_2α_** | 353 | 309 | 2.4 |
| **PGE_2_** | 351 | 271 | 2.6 |
| **PGD_2_** | 351 | 271.01 | 3 |
| **Lipoxin B_4_** | 351 | 221 | 3.1 |
| **15-HETE** | 319 | 219 | 9.8 |
| **11-HETE** | 319 | 167 | 9.9 |

**Table S3. MS/MS calibration and retention time for the AA COX-1 metabolites measured upon whole blood stimulation *in vivo* with AA.**
